# Tumor Organoid and Microenvironment Cocultures: Implications for Basic and Translational Cancer Research

**DOI:** 10.1002/mco2.70741

**Published:** 2026-04-13

**Authors:** Jiajun Yang, Chunliang Cheng, Wenqin Luo, Xingfeng He, Yaqi Li, Xiang Hu, Sanjun Cai, Hai Zou, Shaobo Mo, Junjie Peng

**Affiliations:** ^1^ Department of Colorectal Surgery Fudan University Shanghai Cancer Center, Fudan University Shanghai China; ^2^ Department of Oncology Shanghai Medical College, Fudan University Shanghai China; ^3^ Cancer Institute Fudan University Shanghai Cancer Center Shanghai China; ^4^ Department of Emergency and Critical Care Medicine Shanghai Pudong New Area People's Hospital Shanghai China

**Keywords:** coculture, immunotherapy, organoid, precision medicine, tumor microenvironment

## Abstract

Organoids are innovative three‐dimensional (3D) cellular constructs, offering a unique platform to replicate the architectural and functional complexity of organs and tissues. In oncology, the tumor microenvironment (TME) dictates tumor evolution and therapeutic resistance. Consequently, therapies targeting TME components have emerged as a burgeoning frontier in cancer treatment. However, accurately recapitulating the dynamic, multicellular crosstalk of TME remains a significant hurdle for clinical translation. This review encapsulates the spectrum of current organoid coculture methodologies, ranging from direct coculture and air–liquid interface to advanced microfluidics and 3D bioprinting. These models not only deepen our understanding of the fundamental mechanisms at play in cancer but also evaluate emerging therapeutic modalities, such as antibody–drug conjugates and immunotherapy. By closely mimicking the in vivo tumor milieu, organoid cocultures enhance our ability to predict therapeutic outcomes and pave the way for the development of precision medicine approaches, thereby propelling forward the frontiers of oncology. This review aims to provide a comprehensive overview of organoid coculture models, spanning from construction methodologies to clinical applications. We envision this work serving as a definitive guide for the field, ultimately accelerating the transition from theoretical research to clinical practice.

## Graphical abstract

Organoid technology has attracted significant attention in cancer research. By incorporating tumor organoids with elements of the tumor microenvironment in coculture systems, researchers have gained powerful new tools to unravel the complex interplay that influences tumor progression and therapeutic outcomes.

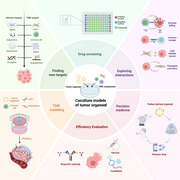

## 1 Introduction

Cancer remains a leading cause of mortality worldwide, yet the development of effective therapeutics is frequently hampered by the high attrition rate in clinical trials. Consequently, there is an urgent need for robust and physiologically relevant in vitro preclinical cancer models [1, 2]. Since the seminal work of Sato et al. [3], who first established mouse gut organoids by culturing Lgr5+ stem cells with a precise blend of growth factors and inhibitors, the utility of organoids in recapitulating the complexity of human tissues has expanded exponentially. Building on this foundation, the same group established in 2011 the first long‐term cultures of human colorectal cancer (CRC) organoids that preserve key histological and molecular features in vitro [4]. Tumor organoids, or patient‐derived organoids (PDOs), are now recognized for their high fidelity to the original tumors, preserving histopathological and genetic features, positioning them as indispensable tools in tumor modeling and precision medicine [5, 6]. The evolution of tumor organoid technology has facilitated the development of sophisticated in vitro models that recapitulate the heterogeneity and complexity of various human malignancies, including CRC [7, 8], advanced prostate cancer [9], bladder cancer [10], breast cancer [11], gastric cancer [12, 13], liver cancer [14], ovarian cancer [15], lung cancer [16, 17], head and neck cancer [18], and cervical cancer [19].

Although tumor organoids successfully reveal tumor heterogeneity and predict responses to chemotherapy [20], conventional cultures remain predominantly reductionist, consisting mainly of epithelial cancer cells while excluding the nonmalignant components of the tumor microenvironment (TME). It is now well‐established that the TME—comprising fibroblasts, immune cells, endothelial cells, and the extracellular matrix (ECM) [21]—plays a pivotal role in tumor progression, metastasis, and therapeutic resistance, particularly in the era of immunotherapy [22]. Consequently, the absence of stromal and immune components in standard organoid cultures limits their utility in studying tumor–stroma crosstalk and evaluating immunotherapeutic responses. To overcome these limitations, there is an urgent need to develop advanced coculture systems that integrate PDOs with autologous or engineered TME components to mimic the native tumor niche more faithfully.

In response to this challenge, recent advancements have yielded a diverse array of strategies to reconstruct the TME in vitro, ranging from simplified submerged cocultures to complex microfluidic “organ‐on‐a‐chip” platforms [23]. Given the rapid evolution of this field, this review aims to provide a systematic overview of the current state of tumor organoid and microenvironment cocultures. Beyond detailing the technical establishment of these platforms, we specifically highlight the dual implications of these integrated systems: their utility in unraveling the fundamental mechanisms of tumor–stroma interactions (basic research) and their critical role in facilitating drug screening and personalized precision medicine (translational research).

In this review, we first outline the conceptual framework of tumor organoid–TME coculture systems and the engineering strategies used to establish them. We then discuss the key mechanistic insights gained from these models, focusing on tumor–stroma interactions. Furthermore, we highlight their translational applications in precision oncology. Finally, we address current challenges, such as standardization, and discuss future directions. Ultimately, we hope this review serves as a practical guide for readers to identify the most suitable coculture models for their specific needs, ranging from basic exploration to clinical translation.

## 2 Conceptual Frame Work of Tumor Organoid–TME Coculture Systems

The conceptual foundation of tumor organoid–TME coculture systems is grounded in the classic “seed and soil” hypothesis. This paradigm postulates that tumor progression is not orchestrated solely by the intrinsic genetic aberrations of cancer cells (the seed) but is critically governed by the surrounding microenvironment (the soil) [24]. TME is a highly complex ecosystem, apart from malignant cells, it includes a diverse array of nonmalignant cell types and noncellular components such as the immune cells, ECM, blood vessels, and soluble molecules [21]. Historically, the TME was overlooked, but current understanding emphasizes its critical role in tumor initiation, progression, metastasis, and response to therapy [22]. The interactions between tumor cells and the TME are dynamic and bidirectional. Tumor cells educate their microenvironment to support malignant growth. This is exemplified by the influence of tumor‐associated macrophages (TAMs) and the reshaping of cancer‐associated fibroblasts (CAFs) through tumor cell‐intrinsic genetic mutations and epigenetic dysregulation [25, 26].

However, accurately mimicking this complex interaction between the “seed” and the “soil” remains a significant challenge in preclinical oncology. Despite significant strides in TME preclinical research and therapy targeting components of the TME, the translation to clinical practice has been inefficient and inconsistent [27]. It may be attributed to the limitations of traditional 2D cancer cell line in vivo and in vitro, failing to fully represent the complex three‐dimensional (3D) architecture and cell interactions present in human tumors [28, 29]. The use of immunocompromised mice in patient‐derived tumor xenografts (PDX) models also limits the study of tumor–immune interactions, and the replacement of human stroma by murine components after several passages further reduces the fidelity of these models to human TME [30].

**FIGURE 1:**
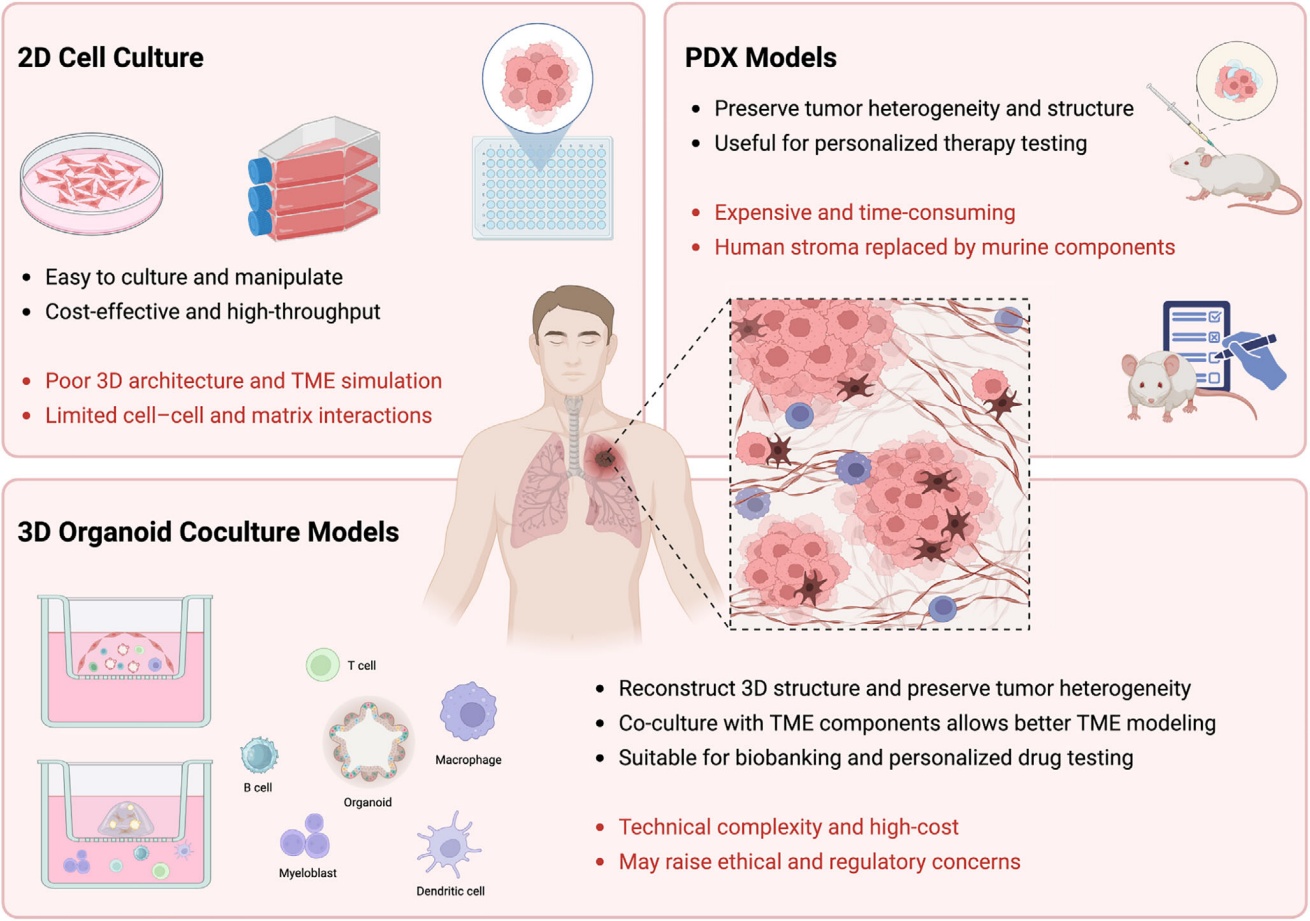
Comparative landscape of 2D cell culture, PDX, and 3D organoid coculture models. Illustration of the advantages and limitations of three commonly used in vitro models. It highlights how each model uniquely contributes to tumor research while also presenting specific challenges related to physiological relevance, complexity, and translational applicability. By BioRender.

To bridge this gap, the development of 3D coculture models within tumor organoids and TME components has become a critical advancement. A conceptually complete coculture system aims to structurally and functionally reconstruct the native tumor niche. Organoid coculture models involve the incorporation of additional cell types into organoid cultures, which may include various components of the TME. This framework involves the integration of three key components: the epithelial compartment (PDOs representing the tumor epithelial), the stromal compartment comprising CAFs, immune cells, and vascular networks, and the ECM, often modeled by hydrogels such as Matrigel, which provides both structural support and biochemical cues. Figure 1 shows the distinct characteristics of these three in vitro models. Compared to traditional 2D cultures and PDX models, these 3D coculture systems more accurately preserve the molecular characteristics, spatial architecture, and signaling dynamics of the original tumor [31]. By enabling both direct cell–cell contact and paracrine signaling, these models provide a robust platform to enhance our understanding of tumor–TME crosstalk [32]. Furthermore, by offering superior predictivity of therapeutic responses, they serve as a critical bridge over the “bench‐to‐bedside” gap, thereby facilitating the successful translation of preclinical findings into clinical benefits [33].

In summary, the conceptual framework of tumor organoid–TME coculture systems represents a fundamental paradigm shift from reductionist approaches to holistic ecosystem modeling. By reuniting the “seed” with its native “soil” in vitro, these models restore the critical signaling networks that define tumor behavior, offering an unprecedented opportunity to decipher mechanism of disease progression and predict therapeutic outcomes. However, translating this biological concept into a functional physical model requires sophisticated fabrication techniques. In the following section, we will systematically examine the diverse engineering strategies employed to construct these complex systems.

## 3 Engineering Strategies for Organoid–TME Coculture

To better mimic the TME, coculture strategies have shifted from simple cellular mixing toward precision assembly. This section evaluates current engineering strategies based on their capacity to control spatial organization and biochemical signaling. We discuss direct coculture without matrix and traditional matrix‐dome models, modular Transwell systems, and high‐precision 3D bioprinting and mirofluidic platforms. Each approach is analyzed by its balance of physiological relevance, experimental throughput, and ability to recapitulate dynamic cell–cell communication.

### 3.1 Coculture Without Matrix

Direct mix‐up suspension is the most straightforward coculture method, in which directly mixing organoids and nonmalignant components in a liquid medium. As organoids may fail to grow without the support of matrix, the coculture model will not last for enough time for observation [34]. Even so, there still are some applications in practice. Direct coculture with autologous organoids offers a reactive platform to enhance tumor‐reactive T cells isolated from patients’ peripheral blood. In this concept of coculture, organoids serve as a catalyst for immune cell differentiation, yielding a pool of immune cells that can be utilized as substrates for subsequent coculture investigations [35]. On the other hand, it can also serve as a model to demonstrate the interaction between the immune system and tumors in some specific situations. In an experiment of engineered exosomes dendritic cell (DC)‐primed vaccine, peripheral blood mononuclear cells (PBMCs) from breast cancer patients were cocultured with autologous PDOs and the vaccine [36]. This direct mix coculture finally validate the vaccine's effectiveness in enhancing DCs and tumor‐reactive CD8^+^T cells activation. To standardize the modeling of immune interactions, Cattaneo et al. [37] established a robust protocol for the direct coculture of tumor organoids with autologous T cells. This framework provides a reproducible platform to evaluate T‐cell‐mediated cytotoxicity and has become a technical benchmark for studying personalized cancer immunotherapy in vitro. Direct suspension coculture of tumor organoids and CAFs in culture medium is uncommon due to the necessity for both of them to reside within the ECM for optimal viability. However, some studies have reported the feasibility of this coculture combination, Dang et al. [38] build suspension culture of CAFs with CRC organoid lasting for 12 days. In summary, despite the loss of long‐term viability without ECM support, “matrix‐free” suspension is a pragmatic choice when the research focus shifts from organoid morphology to the functional activation of cocultured components. In these contexts, the organoid serves primarily as a biological stimulus for immune priming rather than a sustained structural model.

### 3.2 Matrix Dome‐Based Coculture

As the requirement for matrix support in organoid cultures, the coculture method on the basis of Matrigel domes is more commonly used. It offers high flexibility in practical research, as nontumor components of TME can be directly mixed with tumor organoids within the matrix, or added to the surrounding culture medium. Coculture of organoids and immune cell mixed in matrix is one of the common approach, by which Yana's team explore the combined approach of blocking PD‐L1/PD‐1 along with suppressing immunosuppressive MDSCs. For instance, Yana's team explore the combined approach of blocking PD‐L1/PD‐1 along with suppressing immunosuppressive myeloid‐derived suppressor cells (MDSCs) by mixing gastric PDOs with MDSCs in Matrigel [39]. As for the coculture of CAFs and tumor organoids, they are also typically combined and cocultured within a matrix dome containing Matrigel and collagen I gel. Certainly, enhancements can be made to the mixed coculture model. CAFs can be positioned at the periphery within the dome to enveloping organoids to mimic the 3D architecture of tumors in vivo. Coculturing mouse acinar cell organoids with CAFs in this way reveals that fibroblasts prompt acinar‐to‐ductal cell transdifferentiation, and initiate pancreatic cancer [40]. Besides, CAFs can also be seeded on the top the dome to investigate CAF infiltration, mobility, and paracrine interplay between CAFs and tumors. For instance, it contributes to establish coculture system in which colon cancer organoids and CAFs spontaneously organize into superstructures. In the model, CAFs were placed on the surface of PDO‐containing hydrogels and cultured in medium without specific antibiotics and growth factors. The results show that even without typical growth factors, CAFs can still release factors that support CRC PDO growth [41]. On the other hand, incorporating nontumor components into the organoid culture medium, is typically utilized to assess the infiltration capacity of immune cells. For example, pretreated T cells were added to the organoid culture medium to evaluate the infiltration ability of T cells [42]. Moreover, when CAFs are added to the coculture medium in suspension, they initially surround and enter the matrix droplets containing CRC organoid within 2–3 days. Inside the droplets, CAFs organize into stromal tracks that guide the reorganization of the organoids. Ultimately, the organoids merge into “mini tumors” that envelop the CAF tracks [43].

In summary, matrix dome‐based coculture stands as a foundational cornerstone in the field, as it directly evolves from the standard organoid expansion systems. This method faithfully recapitulates the spatial architecture of native human tumors, where epithelial nests are embedded within a dense stromal matrix and sustained by nutrients from the microvasculature. Whether investigating the complex crosstalk between TME components [44] or assessing therapeutic drug efficacy [39], the matrix dome provides a highly biomimetic platform that balances physiological relevance with experimental accessibility.

### 3.3 Transwell‐Based Coculture

Apart from the mentioned coculture techniques, employing Transwell system offers another avenue to establish coculture models for investigating diverse inquiries. Organoids and immune cells can be cocultured without direct contact by employing Transwell inserts. Air–liquid interface (ALI) system is a unique coculture approach which facilitates the growth of PDOs and the preservation of integrated immune elements along with fibroblast stroma. In this method, tumor tissues are finely chopped and suspended in type I collagen, then seeded onto a permeable insert precoated with collagen matrix, which is placed in an outer tissue culture dish containing growth medium [32]. The ALI system is proved to functionally recapitulate the PD‐1/PD‐L1‐dependent immune checkpoint, which shows its potential for evaluation of cancer immune therapy [45]. The rational use of Transwell in ALI has inspired other coculture methods. In the establishment of macrophage–organoid coculture model, a 0.4 µm Transwell was introduced into a 6/24‐well plate, effectively segregating it into two chambers: one designated for macrophages and the other for organoids [46]. In this in vitro setup, macrophages and pancreatic cancer cells (PCCs) were separated into two compartments without direct contact, with the goal of studying soluble chemical signals like cytokines, exosomes, and metabolites that could promote resistance to gemcitabine.

For CAFs coculture, Transwell also provides new methods. In a seminal study investigating the unique subsets of inflammatory fibroblasts and myofibroblasts in pancreatic cancer, Öhlund et al. [47] pioneered 3D coculture systems involving neoplastic pancreatic ductal organoids and pancreatic stellate cells (PSCs) and demonstrated the presence of two phenotypically distinct populations of CAFs within distinct coculture models. In detail, when organoids are combined with PSCs and embedded in Matrigel, direct physical contact activates PSCs, leading to their differentiation into myofibroblastic CAFs (myCAFs) characterized by high levels of αSMA and low levels of IL‐6. These myCAFs tend to localize proximally to the neoplastic cells. Conversely, in the Transwell coculture setup, PSCs are seeded in Matrigel on the upper side of the Transwell membrane, while organoids grow in the lower compartment of the 24‐well plates. This configuration allows for paracrine interactions between tumor organoids and PSCs while preventing direct contact between the two cell types. In this setting, a different population of CAFs known as inflammatory CAFs (iCAFs) emerges. iCAFs exhibit low levels of αSMA but high levels of IL‐6 and are induced by paracrine signaling from the tumor compartment. They are distributed more distantly throughout the tumor. This rational use of Transwell in this study is an excellent for studying CAFs subsets as well as showing the great potential of CAFs and organoid coculture model.

Using Transwell systems smartly allows for the creation of diverse culture methods tailored to experimental needs. In the process of establishing a model for CRC organoids, researchers combined epithelial cells and fibroblasts obtained from the same patient [48]. Initially, they cocultured fibroblasts and PDOs in Matrigel drops to assess their viability in vitro. Subsequently, they embedded fibroblasts in a collagen I gel, coated it with Matrigel, and placed organoids on top to examine whether this coculture model could replicate the histological characteristics of the original tumor because this method closely mimicking the tissue architecture of the colon in vivo. To investigate the communication between stromal fibroblasts and tumor epithelial cells and to facilitate gene expression analysis, researchers adopted a modular approach. Organoids were cultured in Matrigel domes while fibroblasts were cultured in collagen I gels; however, these gels were positioned on opposite sides of a Transwell membrane insert [48].

In summary, Transwell‐based systems enrich coculture methodology by providing a unique dual‐chamber architecture. This physical segregation eliminates confounding effects from direct cell‐to‐cell contact, making it the premier platform for dissecting noncontact‐dependent interactions. It is particularly indispensable for isolating the effects of paracrine signaling, soluble secreted factors like cytokines and chemokines, and extracellular vesicles (EVs), thereby allowing researchers to precisely decipher the long‐range communication networks within the TME.

### 3.4 3D Bioprinting and Microfluidic Devices

In the swift advancement of technology, numerous emerging methods have been deployed to build coculture models, with 3D bioprinting and microfluidics standing out as prime illustrations. 3D bioprinting involves crafting in vitro 3D structural models composed of biological components like cells, proteins, and DNA [49]. 3D bioprinting technology holds promise for organoid coculture by overcoming traditional culture limitations, replicating complex TMEs, and directly constructing 3D coculture models as preset [50]. A bioprinted model is created with separate areas housing gastric cancer PDOs, tumor‐infiltrating lymphocytes (TILs) and mechanically defined alginate–gelatin–basement membrane hydrogel. This setup is designed to evaluate and replicate the migration and functional activation of TILs within the TME, which introduces a new experimental format to evaluate TIL infiltration, motility, and cytotoxicity capabilities [51]. At the same time, 3D bioprinting technology has shown potential in multicomponent coculture in simulated TME [52]. A strategy involving embedded bioprinting, utilizing a finely tuned collagen I‐based bioink, can be employed to produce biomimetic tumor models. These bioprinted breast tumor models effectively recapitulate key aspects of the TME, such as angiogenesis, epithelial–mesenchymal transition (EMT), and invasion [53]. These 3D printed models are advancing rapidly, expected to emulate the TME with greater fidelity and introduce novel tools for translational medicine.

As for microfluidic devices, designed to mimic essential aspects of the TME, they have emerged as innovative and dependable tools. These devices usually include tiny channels, chambers, and compartments, allowing precise management of different environmental conditions like nutrient distribution, oxygen concentration, cellular interactions, pH, and the presence of immune cells or ECM components by manipulation and control of tiny small amounts of fluids [54, 55]. They enable the study of how tumors modulate their TME to evade antitumor immunity and elucidate the mechanisms underlying tumor resistance to immunotherapy. In microfluidic coculture system, organoids derived from patients exhibiting sensitivity or resistance to immunotherapy maintained consistent drug responsiveness in vitro [56]. Moreover, this system facilitated the screening of small molecule drugs aimed at enhancing the effectiveness of PD‐1 monoclonal therapy. It was discovered that combinations of CDK4/6 and TBK1/IKKe inhibitors with PD‐1 monoclonal antibodies bolstered the antitumor immune response [57]. Recently, accurate microfluidic models have been developed, which includes many components of the TME. Microengineered liver cancer organoid‐on‐a‐chip using a coculture model involving mesenchymal stem cells (MSCs), PDOs, and PBMCs is successfully constructed, in which reduces the time required for high‐throughput organoid culture and drug screening and provides more accurate predictions of how patients with Hepatocellular carcinoma (HCC) might respond to common anticancer drugs, particularly immunotherapies like immune checkpoint inhibitors [58]. An innovative patient‐specific lung cancer assembloid (LCA) model using droplet microfluidic technology, employing a microinjection strategy is successfully established. This advanced model enables the prediction of personalized treatments by facilitating the coculture of tumor organoids, immune cells, and CAFs within a microfluidic platform [59]. Moreover, 3D bioprinting can be integrated with microfluidic platforms to generate biomimetic and functional tumor‐on‐a‐chip systems. For example, Yi et al. [60] developed a bioprinted human glioblastoma‐on‐a‐chip by spatially organizing patient‐derived glioblastoma cells, vascular endothelial cells, and decellularized brain ECM in a concentric, compartmentalized architecture. This platform sustained a radial oxygen gradient and recapitulated key structural, biochemical, and biophysical features of native glioblastoma tumors. Importantly, the model reproduced patient‐specific responses and resistance to concurrent chemoradiotherapy and temozolomide, demonstrating its potential as an ex vivo platform for personalized therapeutic screening.

Although 3D bioprinting and microfluidic technologies have great advantages in constructing complex multicomponent coculture models, there are still some problems to be solved. 3D extrusion bioprinting struggles to quickly create small, accurately sized tissue models and achieving uniformity in rapidly preparing tumor assembloids remains a major challenge [49, 61]. This is constrained by current technical limitations. 3D bioprinting must navigate the delicate trade‐off between printing resolution and cellular health. High‐resolution printing often requires narrow nozzles and high extrusion pressures, which subject cells to damaging mechanical stress, thereby reducing postprinting survival rates. Additionally, maintaining the structural integrity of complex biomimetic architectures while ensuring rapid vascularization and nutrient diffusion remains a bottleneck for scaling up these models [62]. As for microfluidic devices, the problem of decreased cell viability and consistency in long‐term cultures, the inability to model the heterogeneity of the entire tumor, and the inability to fully replicate all components of the TME are limitations at this stage [23, 63]. Furthermore, the operational challenges include channel clogging caused by cell aggregates or debris, which can disrupt laminar flow and compromise experiment continuity [64, 65].

In general, emerging coculture methods makes multicomponent coculture accessible and thus additionally offers a more lifelike model for investigating the interactions between tumors and TME, assessing drug effectiveness, pioneering novel therapies, and ultimately advancing the aims of precision medicine.

**FIGURE 2:**
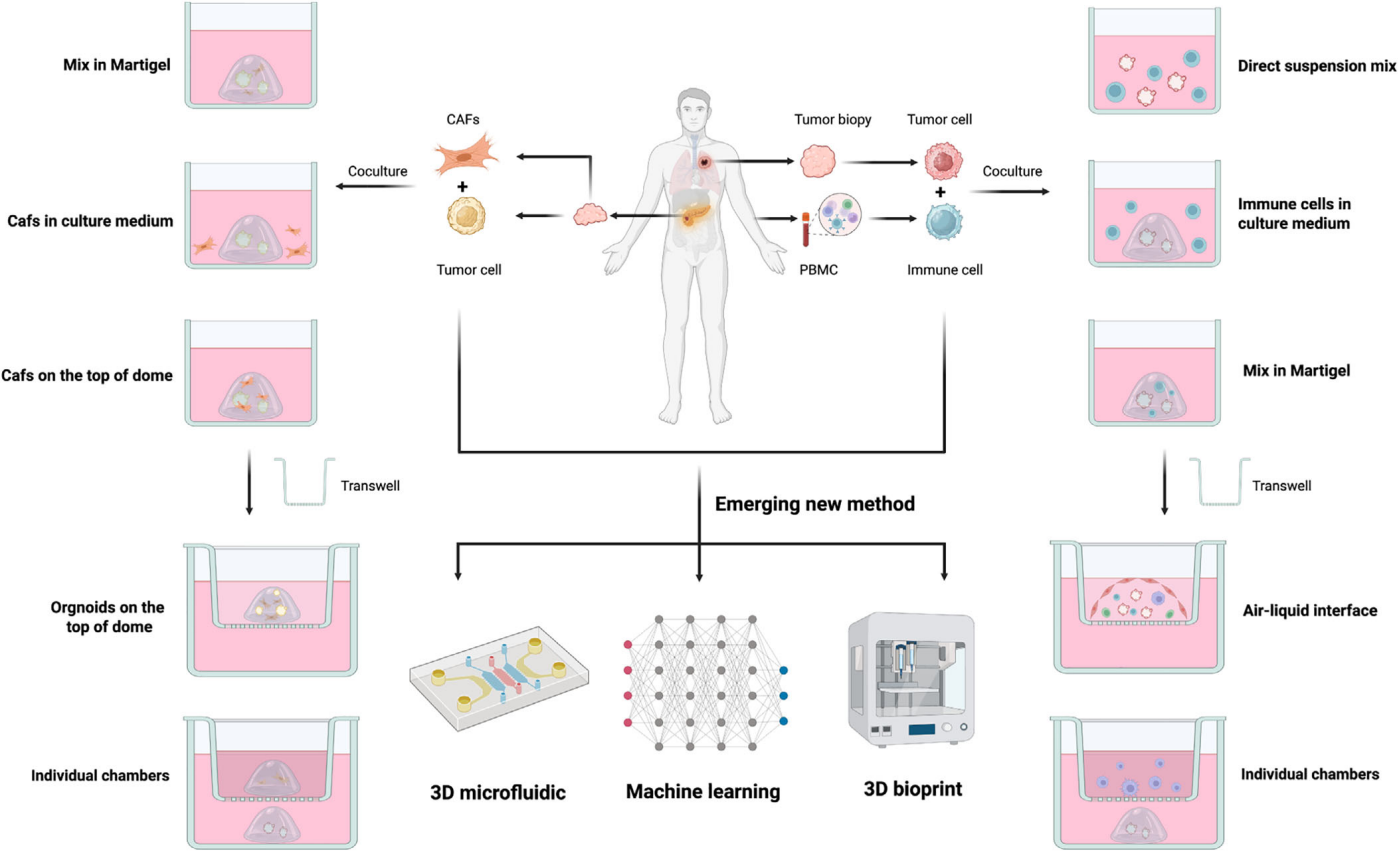
Flowchart for establishing coculture models. Schematic flowchart illustrating the integration of PDOs with cancer‐associated fibroblasts (CAFs) from biopsies or PBMCs for immune‐interaction studies. Diverse coculture methodologies are depicted alongside the incorporation of advanced technologies, including 3D microfluidics, bioprinting, and machine learning, to enhance model complexity and predictive accuracy. By BioRender.

To conclude, the establishment of a variety of coculture modes provides us with a foundational framework for investigating the interplay between the TME components and tumors. Table 1 summarizes different coculture models constructed using various coculture methods. Flowchart for establishing coculture models especially those involving CAFs and immune cells, is presented in Figure 2. From straightforward direct hybrid cocultures to emerging method like microfluidic devices, each approach has its own set of advantages, disadvantages, and applications. The choice of coculture method should be closely related to the experimental design and purpose. Beyond structural assembly, these tailored coculture platforms provide an unprecedented window into the dynamic “crosstalk” between cancer cells and their surroundings. In summary, direct coculture offers a cost‐effective approach for short‐term studies where the long‐term physiological state of the organoids is not the primary focus. Standard methods rely on Matrigel. By incorporating coculture components within the matrix or the surrounding medium, both contact‐dependent and paracrine signaling interactions can be analyzed. Alternatively, Transwell inserts provide a more effective platform for isolating the effects of soluble factors. For more advanced requirements, emerging technologies such as 3D bioprinting and microfluidics allow for the customization of models to meet specific research objectives, such as spatial architecture or fluid shear stress.

**TABLE 1:** Establishment of tumor organoid and TME coculture.

Coculture models	Cell types	Modeling for	References	Year
Direct coculture	Lymphocytes	enhance tumor‐reactive T cell	[34]	2018
	PBMCs and DC vaccine	validate the vaccine are effective in enhancing DC and tumor‐active CD8+ T cells activation	[36]	2022
	CAFs	purpose of histologic observation	[38]	2023
Matrix dome‐based coculture	MDSCs	explore the combined approach of blocking PD‐L1/PD‐1 along with suppressing MDSCs	[39]	2021
	CAFs	explore CAFs prompt acinar‐to‐ductal cell transdifferentiation and initiating pancreatic cancer	[40]	2024
	CAFs	investigate infiltration, mobility, and the paracrine of CAFs	[41]	2021
	T‐cells	assess the infiltration capacity of T cells	[42]	2021
Transwell‐based coculture	Macrophage	study macrophage‐produced soluble chemical signals	[46]	2023
	CAFs	explore the differentiation of CAFs subsets	[47]	2017
	CAFs	mimicking the tissue architecture of the colon in vivo	[48]	2023
3D bioprinting	TILs	evaluate and replicate the migration and activation of TILs	[51]	2023
	Endothelial and CAFs	recapitulate key aspects of the tumor microenvironment	[53]	2023
Microfluidic devices	MSCs and PBMCs	predict how patients with HCC respond to anticancer drugs	[58]	2023
	Immune cells and CAFs	predict personalized treatments by lung cancer assembloid	[59]	2024

Abbreviations: CAFs, cancer‐associated fibroblast; DC, dendritic cells; HCC, hepatocellular carcinoma; MDSCs, myeloid‐derived suppressor cells; MSCs, mesenchymal stem cells; PBMCs, peripheral blood mononuclear cells; TILs, tumor‐infiltrating lymphocytes.

In the following section, we will delve into how these models have yielded critical mechanistic insights into tumor progression, immune evasion, and therapeutic resistance.

## 4 Mechanistic Insights Gained From Organoid–TME Cocultures

### 4.1 Overview

**FIGURE 3:**
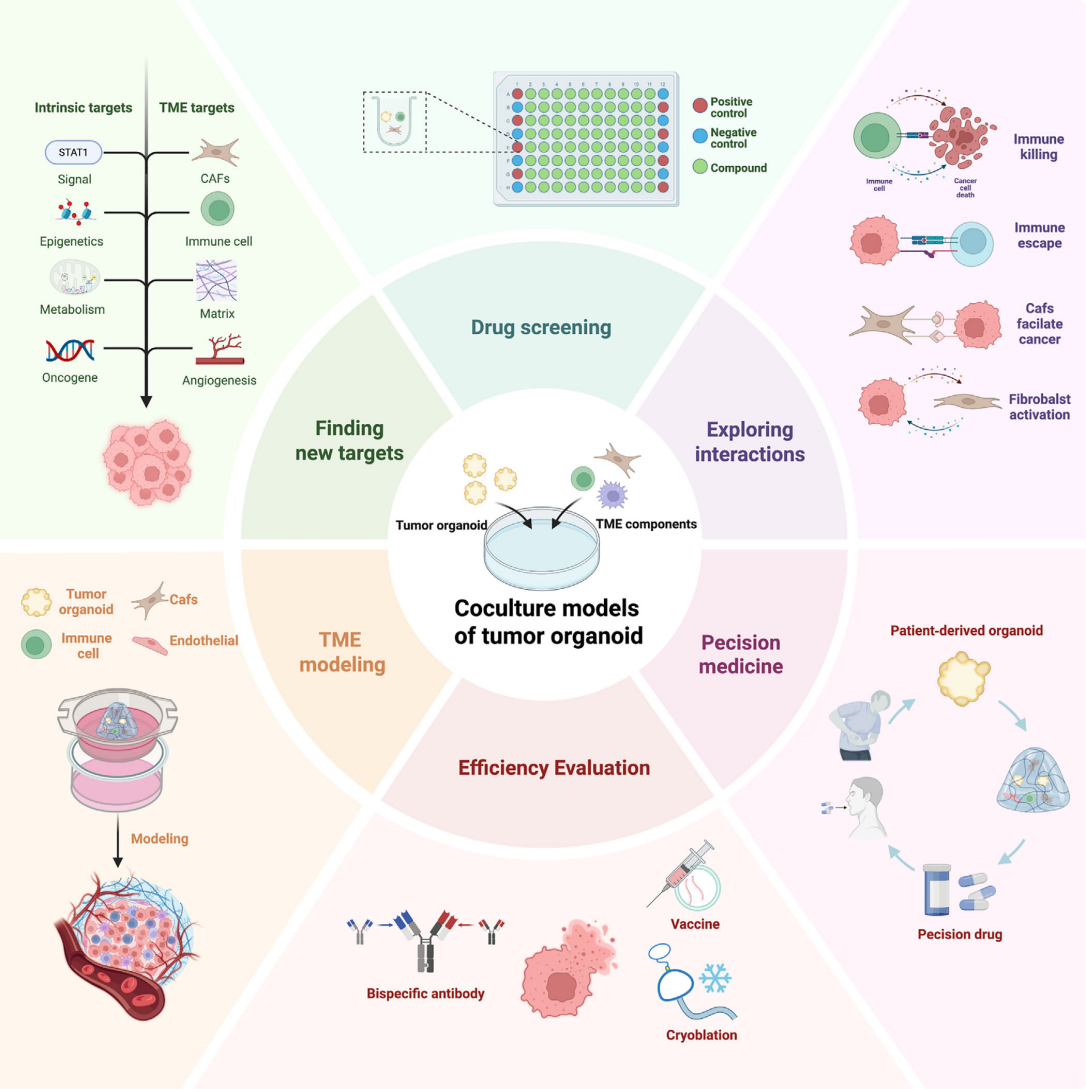
Schematic diagram of applications for tumor organoid coculture models. Coculturing tumor organoids with TME components enables simulation of the complex human TME, exploring interactions between tumors and TME components to identify new therapeutic targets for antitumor drugs. Furthermore, coculture models provide optimal experimental materials for drug screening and effective evaluation platforms for emerging cancer therapies. Due to the patient‐specific nature of patient‐derived organoids, these models can also deliver personalized treatment strategies, driving advancements in precision medicine. By BioRender.

Figure 3 shows the schematic diagram of applications for tumor organoid coculture models. A significant application of organoid coculture is the exploration of complex interactions among components in TME. The intricate communication between tumor cells and other TME components has emerged as a research focal point [66]. These interactions are mediated through direct cell‐to‐cell contact via receptor–ligand binding and gap junctions, as well as matrix mechanical signaling that translates physical stiffness into cellular cues [67, 68]. Additionally, secretory pathways—including paracrine signaling via cytokines and EV‐mediated crosstalk play pivotal roles [69]. Furthermore, metabolic coupling enables the exchange of nutrients and metabolites, effectively reprogramming the tumor's energetic landscape [70].

This section focuses on the major constituents of the TME—namely, the immune condition, stromal components, vasculature, and microbiota—with immune cells and CAFs serving as central themes of the discussion. In general, the immune condition in TME presents as chronic and damaging inflammation and tumor immunosuppressed [21]. The immune suppression is caused by the dysregulation of various immune components in the TME including dysfunctional CD8^+^T cells, regulatory T cells (Tregs), multiple TAM subsets coexistence and so on [71]. Moreover, CAFs were thought to be involved in the remodeling of ECM, while recent research has shown that CAFs with enormous plasticity are composed of different subpopulations and exert multifaceted and contradictory roles in the TME by not only remodel the ECM and alter mechanical characteristics but also influence the cancer cells, angiogenesis, and immunomodulatory in the TME [21]. Establishing TME–organoid coculture is to reveal the complex relationship and give implications for translational research. Of course, we acknowledge that the TME has other components like adipocytes and even microbiota, we also discussed their new findings under coculture conditions. The following examples of cocultured cell types illustrate recent findings on organoids coculture in TME interaction studies.

### 4.2 T Cells

T cells are key components in both the overall human immune system and within the TME. For example, CD8+ T cells serve as effector cells in the antitumor immune response, employing mechanisms such as granzyme and perforin‐mediated apoptosis, as well as FASL‐FAS‐mediated cell death, to destroy target cells [71]. However, many different CD8+ T‐cell states can be found in TME, such as dysfunctional or exhausted phenotype CD8+ T cell [72]. T‐cell exhaustion refers to a general term for the dysfunctional states observed in antigen‐specific CD8+ T lymphocytes, initially described in the context of chronic viral infections, where these cells persist but fail to eliminate the pathogen [73, 74]. Later, it was found that in cancer, CD8+ T exhaustion also occurs, making these T cells similarly unresponsive to tumor cells. The onset of exhaustion is associated with the surface expression of coinhibitory receptors, which regulate CD8+ T‐cell function [75]. For this mechanism, the emerging immune checkpoint blockade aims to unleash CD8+ T‐cell responses against cancer.

In the coculture model involving T cells and tumors, implications for crosstalk between T cells and tumor have come to light. Using a coculture system of CD8+ T cells and CRC organoids, Sui et al. [42] discovered that DKK1 directly suppresses CD8+ T‐cell activation. Furthermore, DKK1 neutralization was found to enhance the tumor response to PD‐1 blockade in dMMR/MSI CRC cells.

CD4+ T cells are also gaining attention in tumor immunity. Cholangiocarcinoma (CCA) organoids were cocultured with either PBMCs or T cells to emulate immune cytotoxicity against CCA [34]. The cytotoxic effects were observed to occur through direct cell–cell contact and the release of soluble factors, thus confirming the pathways involved in T‐cell‐mediated tumor cell killing. This study underscores that the interactions between T cells and tumors is intricately linked to both tumor heterogeneity and the complex immune factor microenvironment. One soluble immune regulator is the acute phase protein serum amyloid A (SAA), which is proved to be secreted by cancer stem cells and drive type 2 immunity [76]. To explore the biological impact of SAA signal in the context of antitumor immunity, an autologous ex vivo lung cancer organoids–PBMCs coculture model is established. Subsequent trials based on the model proved that SAA signal restricts the polarization of type 1 immunity while promoting polarization toward type 2 immunity which partially mediated by the induction of IL‐33 secretion in malignant cells. This hampers the cytotoxic effects of T cells on malignant cells, a process that was mediated by the PD‐1 antibody in ex vivo coculture model [77].

In addition to interactions with soluble immune factors, cancer cells can acquire T‐cell marker proteins through trogocytosis, an interesting process where cancer cells transfer membrane proteins from donor cells to their own surfaces. Unlike other endocytic mechanisms, trogocytosis preserves the cellular localization and functions of the transferred membrane proteins [78]. Through this mechanism, tumor cells induce functional changes, particularly immunosuppression, within the TME. In an in vitro colon organoid coculture with CD4+ T cells, the generation of cancer cells containing both tumor and T‐cell markers, specifically CD4, is observed. These findings indicate that tumor cells are capable of acquiring proteins from contacted CD4+ T cells, while normal colonic epithelial cells do not exhibit this ability [79]. It gives a new insight of therapeutic approaches targeting trogocytosis.

The intestine harbors a significant population of distinctive T cells referred to as intraepithelial lymphocytes (IELs). Positioned closest to the intestinal epithelial cells (IECs), IELs reside within the intestinal epithelial monolayer. These cells encompass not only conventional CD4+ or CD8+ T cells, but also unconventional γδ T cells or CD4‐CD8αβ‐TCRαβ+ T cells [80]. A new coculture setup involving IELs and intestinal tumor organoids derived from small intestinal tumors has been developed to explore the role of IELs in surveilling IECs and excluding tumor cells. In a separate culture experiment, it was observed that the antitumor immune response of IELs against intestinal tumor organoids relies on cell‐to‐cell contact and is associated with the CD103/E‐cadherin signal pathway. Additionally, coculture models demonstrate the indispensability of each IEL fraction for an effective antitumor immune response [81]. Taken together, the novel mechanisms of intercellular communication uncovered by these coculture models hold great promise as potential targets for next‐generation T‐cell‐based cancer immunotherapies.

### 4.3 B Cells and Tertiary Lymphoid

Compared to the extensive body of research on T‐cell coculture models, B‐cell‐based tumor organoid systems remain relatively rare. This scarcity is largely attributed to the traditional view that B cells, primarily sequestered in lymph nodes, exert their antitumor effects through systemic antibody secretion rather than direct cytotoxic cell‐to‐cell contact [82]. However, the emergence of tertiary lymphoid structures (TLS) has shifted this paradigm, becoming a focal point in cancer immunotherapy. Unlike primary and secondary lymphoid organs, which are innate and permanent, TLS are organized aggregates of immune cells that form de novo within nonlymphoid tissues, such as the tumor. B lymphocytes are the cornerstone of these structures. Within the TLS, B cells can differentiate into antibody‐secreting plasma cells, enabling the localized production of tumor‐specific antibodies [83]. Clinical evidence overwhelmingly suggests that a high density of TLS correlates with superior clinical outcomes and enhanced responsiveness to immune checkpoint inhibitors, such as PD‐1 blockades [84, 85].

Several research have confirmed the feasibility of integrating B cells into tumor organoid coculture system. First, the ALI method allows PDOs to retain endogenous B and T cells within their native stroma. As demonstrated by Neal et al. [32], this undissociated approach maintains TLS‐like aggregates without exogenous cytokines, offering a high‐fidelity replica of the original tumor–immune landscape. Second, for models utilizing peripheral B cells, biochemical supplementation has proven effective. Following the protocols of Wagar et al. [86], the addition of CD40L and IL‐21 successfully mimics T follicular helper (Tfh) signals, driving B‐cell activation and differentiation into antibody‐secreting plasma cells.

In summary, the integration of B cells and the recapitulation of TLS within organoid coculture systems represent a burgeoning frontier in immuno‐oncology. We anticipate that these coculture models will proliferate in the near future, offering unprecedented insights into the complex roles of B cells within the TME. Ultimately, these platforms will pave the way for identifying novel therapeutic targets and optimizing personalized immunotherapy strategies.

### 4.4 Macrophage

TAMs in the TME are traditionally classified into M1 and M2 subtypes. M1 macrophages are generally associated with antitumor activity due to their secretion of proinflammatory cytokines, while M2 macrophages contribute to tumor progression through their anti‐inflammatory functions [87]. Whereas, recent studies on TAM suggest that their differentiation within the TME represents a continuous spectrum rather than being limited to distinct M1 or M2 polarization states [88]. This highlights the complex link between macrophage differentiation and tumors, with the coculture model aiding new discoveries.

In 2012, Muller's team shows that macrophages can be effectively incorporated into coculture systems of murine or human skin squamous cell carcinoma, enabling the study of macrophage activation toward a tumor‐promoting phenotype [89]. In coculture systems, macrophage polarization can be directed toward the M1 or M2 phenotype by supplementing the medium with recombinant IFN‐γ and LPS or IL‐4, respectively. When macrophages were stimulated with IL‐4, it led to basement membrane degradation, increased collagenase activity, and higher levels of MMP‐2 and MMP‐9, ultimately promoting tumor cell invasion. Notably, after 3 weeks of coculture with tumor cells, macrophages spontaneously shifted to an M2 phenotype even without IL‐4 stimulation. Besides, in 2023, Jiang et al. [46] developed an in vitro coculture model to investigate the interaction between PCCs and macrophages, utilizing 0.4 µm Transwell inserts. Their findings revealed that coculturing with macrophages heightened gemcitabine resistance in PCC organoids, primarily by reducing gemcitabine‐induced apoptosis. Mechanistically, macrophages enhance the stemness of PCCs, and enabling resistance to gemcitabine treatment through the CCL5/AKT/Sp1/CD44 signaling pathway.

### 4.5 MDSCs and Dendritic Cell

MDSCs are pathologically activated neutrophils and monocytes which are closely associated with poor clinical outcomes in cancer [90]. In humans, there are two separate subsets of MDSCs: monocytic‐MDSCs (M‐MDSCs) and polymorphonuclear (or granulocytic)‐MDSCs (PMN‐MDSCs) [91]. PMN‐MDSCs are probable contributors to tumor expansion from a relevant study. In pancreatic cancer organoid coculture system, PMN‐MDSCs suppress the proliferation and effector function of CD8+ T cells, and thereby potentially compromising the effectiveness of anti‐PD‐1/PD‐L1 treatment [92]. Similarly, gastric cancer cocultures also demonstrated that organoids expressing PD‐L1 exhibited resistance to nivolumab in vitro when PMN‐MDSCs were present [39]. These studies hold significant implications for combining MDSC depletion with immunotherapy in addressing drug‐resistant tumors. An illustrative case demonstrates that employing Cabozantinib to target MDSCs depletion in conjunction with immune checkpoint blockade leads to antitumor effects and regression in castration‐resistant prostate cancer [93].

DCs may play a crucial role in the antitumor immune responses. In their immature state, DCs traverse the body, monitoring for pathogens. Upon activation, they mature and migrate to lymph nodes, where they present antigens to T lymphocytes, thereby triggering an adaptive immune response [94, 95]. Importantly, mature and immature DCs exhibit distinct characteristics, behaviors, and functions. Immature DCs are more adept at engulfing tumor cells, whereas mature DCs excel in antigen presentation and the activation of T cells [96]. Subtil et al. [97] conduct coculture metastatic CRC organoid with DCs to explore their interplay. By retrieving DCs from the collagen scaffold and evaluating their phenotype after interaction with tumor organoids, the study revealed alterations in the expression of key molecules such as costimulatory (CD86), antigen presentation machinery (HLA‐DR), and coinhibitory (PD‐L1) markers in DCs. Furthermore, their ability to activate T cells was impacted by the interaction with tumor organoids. These observations suggest that tumors may induce a shift or retention of DCs in an immature state, associated with immune tolerance and protumorigenic effects. Moreover, these understanding could pave the way for identifying potential targets and biomarkers to design interventions specific to DC subsets.

### 4.6 CAFs

**FIGURE 4:**
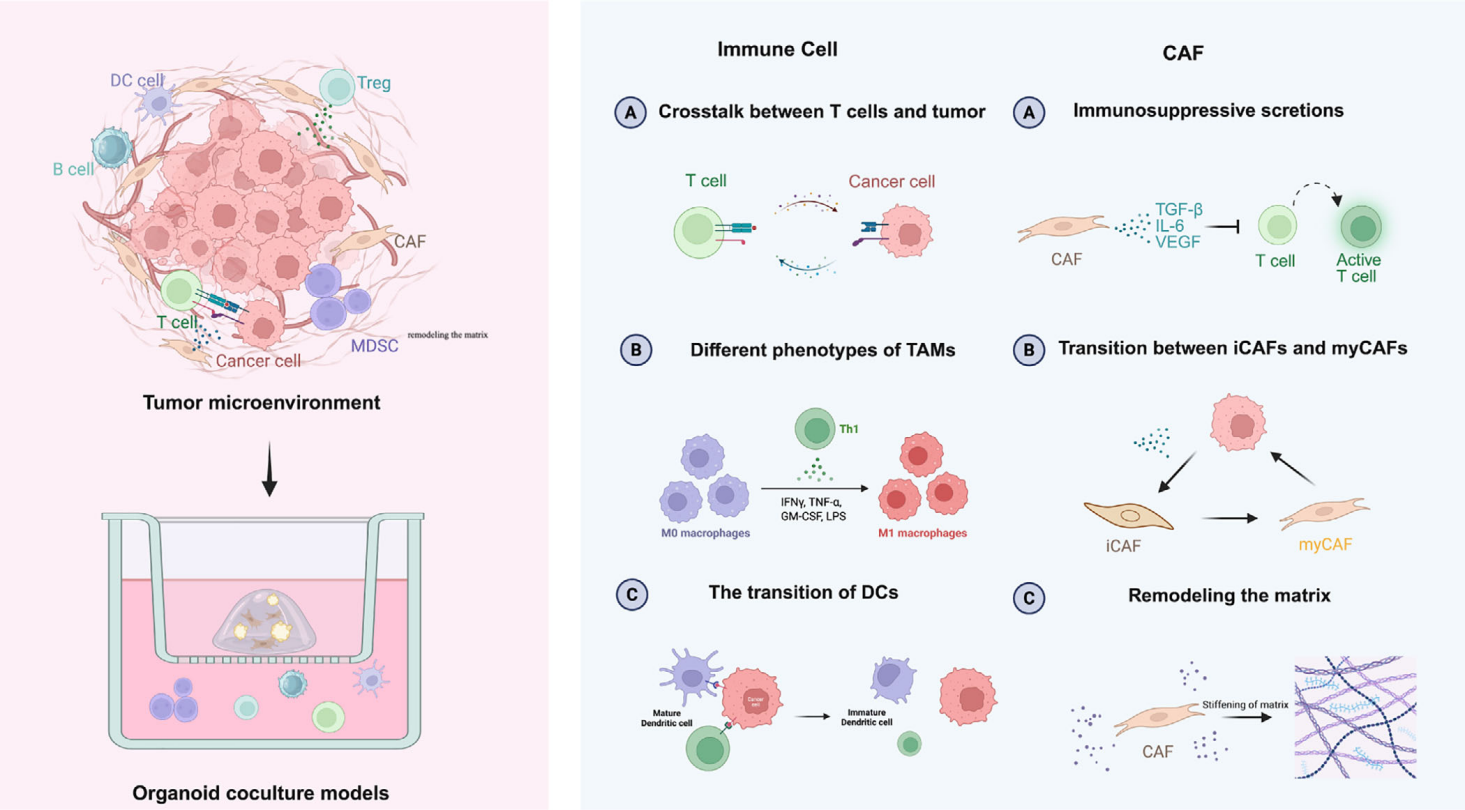
Organoid coculture models are used to recapitulate the complex tumor microenvironment in patients. Immune coculture systems enable detailed exploration of the interactions between tumor cells and key immune components, including T cells, macrophages, and DCs. CAFs coculture models further enhance our understanding of the functional roles and regulatory mechanisms of CAFs within the tumor microenvironment. By BioRender.

The bidirectional interaction between CAFs and tumor cells involves diverse mechanisms including soluble molecules, direct cell–cell contact, matrix remodeling and epigenetic alterations, all of which collectively influence the TME [98, 99]. The 3D organoid coculture model stands out as an effective tool for investigating the relationships, as it offers superior preservation of secretion content and maintains the intricate 3D spatial relationship between CAFs and tumors compared to conventional 2D models [100]. Figure 4 shows that coculture with immune cells and CAFs allows studying interactions among TME components.

The tumor influences CAFs, while CAFs, in turn, also affect the tumor, forming a bidirectional regulatory relationship. The emerging gene analysis technology with coculture models provides a new analytical framework for the relationship between CAFs and tumor cells. CRC‐derived organoids and patient‐matched fibroblasts are cocultured to explore the gene expression patterns [48]. Among the induced genes in CAFs upon coculture, IL6, ICAM1, and CXCL14 stand out, as they promote cell invasion and transmigration. This suggests that tumor cells are instructing CAFs to produce specific molecules, which in turn enable tumor cell propagation and invasion. Analysis of bulk RNA‐seq data in this study unveils gene expression patterns resembling those found in primary tumors. Genes like MUC5AC, IFI6, IFI27, SERPINA1, LYPD8, and CHP2 are notably expressed predominantly in organoids cocultured with fibroblasts, not in monocultures, suggesting a significant influence of CAFs on tumor gene expression. The findings indicate a mutual influence between CAFs and tumors on each other's gene expression, highlighting the promising potential of coculture system for preclinical models.

Precisely designed coculture models reveal distinct populations of iCAFs and myCAFs in pancreatic cancer [47]. Additionally, subsequent studies have revealed that these two CAF phenotypes are not terminal states of differentiation. They possess the ability to transition between each other [101, 102]. Studies based on coculture models have revealed mechanisms underlying the transition between iCAFs and myCAFs. A recent study has defined the critical role of the Prrx1 transcription factor in regulating CAF activation, enabling a dynamic transition between dormancy and activation. In this research, coculture models suggest that Prrx1‐expressing fibroblasts stimulate hepatocyte growth factor signaling, thereby promoting EMT in pancreatic tumor cells, leading to increased invasion and metastasis of pancreatic tumors [103]. Besides, hypoxia can induce more iCAFs phenotypes through HIF‐1α and cytokines secreted by pancreatic tumor cells, thus promoting tumor progression. Schwörer et al. [104] found that PSCs cocultured with PDAC organoids in Matrigel became quiescent and acquired iCAFs state features. Hypoxia alone increased iCAFs marker expression in PSCs to levels similar to organoid cocultures. Additional hypoxia exposure further intensified the iCAFs state in PSCs. These inspired us to target the factors influencing the CAFs phenotype like Prrx1 or HIF‐α with the aim of switching tumor‐promoting CAFs into tumor‐suppressing ones, ultimately leading to tumor suppression.

Apart from the effect of the tumor on phenotype of CAFs, after coculture, the CAFs also have an effect on tumor. Paracrine‐mediated interactions between CAFs and tumor cells have been revealed in many studies [105]. The observation that CAFs enhance the growth or differentiation of tumor organoids in coculture systems has been consistently documented across various established models, including liver cancer organoids [106], CRC [48], and pancreatic ductal adenocarcinoma [107]. Parte et al. [40] revealed that CAFs formed strong associations with acino–ductal/organoid structures, suggesting their capacity to induce transdifferentiation of PDAC. Culture medium produced by CAFs achieved similar acino–ductal cell transdifferentiation effects, indicating that the effect is at least partially paracrine dependent. Subsequent proteomics and RNA sequencing analyses unveiled that CAF‐secreted LAMA5 stimulates ITGA4 expression, thereby fostering acino–ductal transdifferentiation through the STAT3 signaling axis.

Exosomes serve as a non‐negligible pathway for intercellular communication [108]. Significantly, microRNAs transported by EVs from CAFs contribute to drug resistance [109]. In a research basing on coculture models, it was discovered that CAF‐P‐derived EVs contained elevated levels of hsa‐miR‐876‐3p. This miRNA was found to downregulate GATA1 expression in cancer cells. Consequently, the repression of GATA1 led to the inhibition of its target gene, IGFBP3, culminating in cisplatin resistance of oral squamous cell carcinoma (OSCC) [110]. Therefore, this suggests potential strategies for combating tumor drug resistance through the modulation of EVs using specific antagomirs or mimicking miRNAs.

CAFs can also influence the TME through metabolic processes. Organoid coculture model was used to investigate whether parallel CAFs could promote the stem cell‐like properties of OSCC [111]. Follow‐up experiments basing on coculture models revealed that CAFs likely serve as the primary suppliers of lactate. Meanwhile, CD44+ cancer stem cells appear to be the principal consumers of lactate. The interplay of lactate metabolism between these cell types contributes to the progression of tumor organoids.

Besides, CAFs can also engage with tumors through direct cell–cell contact and matrix remodeling. One instance is that CAFs promote invasion of early stage colorectal cancers (T1CRC) organoids into the matrix [38]. Interestingly, this enhanced invasion behavior of T1CRC seemed to rely on direct cell–cell contact rather than paracrine signaling. This was evident as CAFs did not produce differential effects on the invasion of T1CRC organoids when separated by a permeable membrane. The heightened invasion capability of organoids could potentially be attributed to the upregulation of CD44 expression facilitated by direct cell–cell contact. On the other hand, CAFs influence TME by remodeling the matrix. Coculture models shows that CAFs have the capability to reshape the fibrotic environment in PDAC, transforming it into a notably stiffer state through crosslinking, a process dependent on lysyl‐oxidase [112]. In detail, CAFs are initially activated by collagen‐I in the matrix, causing rapid stiffening of matrix. The stiffer matrix activates CAFs further, leading to a hyperactivated state characterized by matrix shrinkage and increased YAP1 expression. This prompts CAFs to produce exosomes, ultimately leading to drug resistance in PDAC. These explorations based on coculture models revealed the remodeling pathway of CAFs to matrix, and inspired us to view matrix as a potential target.

In addition to exploring the crosstalk between CAFs and tumor cells as previously discussed, researchers have employed CAFs coculture models to study chemoresistance. Certain antitumor drugs exhibit efficacy in 2D culture settings but falter in clinical trials, potentially due to the absence of stromal components [113]. Coculturing CAFs with tumor cells serves to compensate for this deficiency, making drug screening outcomes more closely with clinical practice. Currently, a number of CAFs coculture models exhibiting clinically similar drug resistance have been developed, such as pancreatic ductal adenocarcinoma [112, 114], CRC [115], and liver cancer [106]. On the one hand, these models offer a drug screening platform that closely resembles the clinical state. On the other hand, they hold promise for screening personalized drugs using PDOs, thereby advancing precision medicine.

Overall, organoid coculture systems effectively recapitulate the bidirectional crosstalk between CAFs and tumors, encompassing paracrine signaling, metabolic coupling, and physical matrix remodeling. By preserving CAF phenotypic plasticity and spatial architecture, these models reveal mechanisms of stroma‐mediated invasion and chemoresistance that are often lost in 2D cultures. Consequently, these high‐fidelity platforms serve as a robust tool for identifying novel stromal targets and advancing personalized medicine.

### 4.7 Extracellular Matrix and Blood Vessels Endothelial

ECM stands as the principal noncellular stromal element within the TME [116]. The remodeling of ECM is closely related to the progression of tumor [117]. Matrigel serves as the predominant substitute for ECM in the majority of organoid coculture models. While Matrigel has encountered problems such as an unclear source of ingredients and batch‐to‐batch variation [118]. Hence, it cannot entirely replicate the complex and tissue‐specific ECM. Several novel biomimetic hydrogels are currently in development, aiming to replicate the intricate structure of collagen found in the ECM [119, 120]. With the advancements in nanotechnology, numerous artificial hydrogels are being developed as potential alternatives to Matrigel. Poly(ethylene glycol) (PEG)‐based hydrogels offer high tunability in mechanical stiffness and ligand functionalization, enabling precise control over cell–matrix interactions; however, their bioinert nature necessitates additional biochemical modification to support cell adhesion and signaling [121]. Polylactide‐co‐glycolide (PLG) hydrogels provide favorable biodegradability and mechanical strength, but their degradation products may alter local pH and affect long‐term cell viability [122]. Self‐assembling peptides hydrogels closely resemble the nanofibrous structure of native ECM and exhibit excellent biocompatibility, although their relatively limited mechanical robustness and higher cost currently restrict widespread application [123]. Besides, tissue‐specific matrix can also be obtained by decellularized technique, a method removing cells from organs or tissues while maintaining the structure and composition of the ECM [124]. Although decellularized matrices offer superior biological relevance, their variability, limited scalability, and challenges in standardization remain obstacles for routine use in organoid coculture platforms.

Blood vessels are also an integral part of the TME [125]. Angiogenesis facilitates tumor growth by creating new blood vessels that act as pathways to fulfill the increasing metabolic requirements of the expanding tumor [126, 127]. In recent years, there has been significant exploration into the use of organoid–endothelial coculture models. These models offer valuable insights into angiogenic signaling pathways and serve as effective tools for screening antiangiogenic drugs [128, 129]. In addition to angiogenesis, the role of endothelial cell‐mediated angiocrine is increasingly recognized as pivotal in tumor progression [130]. Lim et al. [131] developed HCC–endothelial coculture models, which involved cultivating HCC patient‐derived xenograft (PDX)‐derived organoids alongside endothelial cells. This model mimics the crosstalk between HCC and endothelial cells. The findings from the coculture models demonstrate the upregulation of MCP‐1, IL‐8, CXCL16, and tumor necrosis factor (TNF) signaling pathways, implying that angiocrine signaling within HCC fosters an inflammatory microenvironment that could impact the recruitment and activation of immune cells. This provides insights into the understanding and target of the angiocrine signaling.

### 4.8 Adipocytes and Neural Elements

Recent evidence underscores the dynamic role of adipocytes in the TME, challenging the archaic view of them as simple fuel containers. In the proximity of invasive tumors—especially within the bone marrow or breast—adipocytes transition into a protumorigenic state known as cancer‐associated adipocytes (CAAs) [132]. By releasing a specific repertoire of signaling molecules, CAAs orchestrate significant microenvironmental remodeling, which provides the metabolic and physical support necessary for cancer progression and metastasis [133, 134].

In a coculture model of colon cancer organoid and adipocyte, direct contact with adipocytes was found to facilitate fatty acid transfer, which fueled tumor organoid growth. Notably, this lipid‐dependent metabolic interaction drove the tumor organoid toward a stem‐like state, evidenced by the suppression of differentiation markers (*Sis*, *Muc2*) and the concurrent enrichment of Wnt target genes such as *Lgr5* and *Cd44* [135]. Besides, Mertz et al. [136] developed a geometrically inverted organoid coculture system containing a primary adipose core enveloped by a mammary epithelial layer to investigate the role of the microenvironment in breast cancer invasion. They reported that triple‐negative MDA‐MB‐231 cells exhibited superior invasive capacity compared to luminal MCF7 cells, a process accompanied by significant collagen accumulation in the invaded stroma. Notably, targeting this fibrotic response with pirfenidone effectively reduced tumor cell invasion, underscoring the critical interplay between adipocytes, matrix remodeling, and cancer progression. In conclusion, these models and findings provide novel insights into the interactions between tumors and adipocytes.

As for neural elements, high‐grade malignancy in gastric cancer is frequently accompanied by perineural invasion (PNI), a pathological feature strongly associated with dismal clinical outcomes [137]. While early concepts viewed nerves solely as passive conduits facilitating tumor spread, emerging evidence identifies the ENS as an integral and active component of the TME [138]. This neuro‐neoplastic crosstalk extends beyond physical migration, involving the release of neurotransmitters and neurotrophic factors that actively fuel tumorigenesis and metabolic reprogramming [139]. Recent investigations utilizing hESC‐derived ENS coculture models have elucidated a critical metabolic dimension of neuro‐neoplastic crosstalk. By employing the G‐baToN system, researchers demonstrated that enteric neurons influence gastric cancer organoids predominantly through paracrine signaling rather than physical juxtaposition. Notably, transcriptomic profiling revealed that these neuronal secretomes are sufficient to drive extensive metabolic rewiring in tumor cells, characterized by the upregulation of fatty acid synthesis genes like ACACA and the activation of cholesterol homeostasis pathways, thereby fueling tumor progression independent of direct cell–cell contact [140].

### 4.9 Microbiota

The view of tumors as sterile environments has been challenged by robust evidence of a diverse microbial landscape within the TME [141]. Incorporating bacteria, viruses, and fungi, this intratumoral microbiota is now recognized for its capacity to infiltrate tumor and immune cells, thereby reshaping metabolic profiles and signaling cascades [142]. Rather than being incidental bystanders, these microorganisms are active modulators of cancer progression and host immunity [143]. Consequently, understanding these microbial–host dynamics is becoming essential for optimizing clinical outcomes, as they fundamentally alter the effectiveness of modern oncological treatments like immunotherapy [144].

The establishment of microinjection techniques allows for the direct delivery of microorganisms into the apical chamber of 3D organoids, thereby validating the viability of this organoid and microbiota coculture system [145]. A landmark study by Pleguezuelos‐Manzano et al. [146] utilized this approach to demonstrate that chronic exposure of intestinal organoids to *pks+ E. coli* induces a specific mutational signature. This signature, characterized by DNA alkylation on adenine residues, was subsequently validated in a significant proportion of CRC patients, thus establishing a mechanistic link between chronic colibactin exposure and human CRC development. Beyond driving initial oncogenic mutations, tumor‐resident microbes have also been implicated in modulating the functional plasticity of malignant cells. A prominent example is the work of Fu et al. [147], who expanded the application of tumor organoid cocultures to investigate how intracellular bacteria influence metastatic potential. By establishing a system where dissociated breast cancer cells were coseeded with fluorescently labeled bacterial strains, they observed that specific genera, such as *Staphylococcus* and *Lactobacillus*, could actively invade and persist within the host cell cytosol. To decode the functional consequences of this intracellular residency, the authors employed single‐cell RNA sequencing on bacteria‐positive tumor cells isolated from the organoids. Their findings revealed that these microbial “guests” actively reprogram the host transcriptome, specifically enhancing cellular resistance to fluid shear stress and promoting metastatic colonization.

In summary, the research detailed in Section 4 highlights the unique capability of organoid coculture systems to unravel the complex nexus of signaling cascades, metabolic shifts, and mechanical remodeling that fuels tumor progression. By deciphering these dynamic interactions, these models have not only expanded our biological knowledge but also pinpointed exploitable vulnerabilities within the TME. Crucially, the high fidelity with which these systems mimic in vivo complexity is the key to their translational value. Building on this foundation, the subsequent section will bridge the gap between mechanistic understanding and clinical application, examining how TME‐integrated organoids are currently utilized for high‐throughput drug screening, immunotherapy assessment, and the advancement of precision oncology.

## 5 Translational Applications in Precision Oncology

In recent years, the management of tumors has evolved significantly, transitioning from conventional methods like radiotherapy and chemotherapy to cutting‐edge approaches such as immunotherapy, cell therapy, and combination therapy [148]. However, the therapeutic efficacy of these modalities is intrinsically modulated by the TME. The translation of mechanistic insights into tangible clinical benefits hinges on the ability of preclinical models to predict patient‐specific responses with high accuracy. Unlike traditional 2D cultures or PDX, TME‐integrated organoids offer a physiologically relevant platform for validating effect of TME‐related drug and explore new immunotherapies, which will eventually facilitate the translation of promising treatments from the laboratory to the clinic.

### 5.1 High‐Throughput Drug Screening and Biobanking

The establishment of living biobanks derived from patient biopsies has revolutionized preclinical drug discovery [149]. These biobanks now enable high‐throughput screening (HTS) of hundreds of compounds, allowing for the identification of effective therapeutic agents within a clinically relevant timeframe [11]. The comprehensive overview of tumor–stroma mechanisms presented in Section 4 lays the theoretical foundation for therapeutic strategies targeting the TME. Monoculture tumor organoid biobanks are insufficient for screening drugs that target TME components. Therefore, establishing coculture models for HTS is essential for cancer immunotherapy and stroma‐targeted therapies.

Integrating high‐throughput automation with biologically relevant TME models, Mason et al. [150] executed a massive screen of about 36,000 compounds in a PDAC–stroma coculture system. This robust platform distinguished itself by identifying GNF‐5, an agent that functionally reprograms stromal cells to a protective myCAF state. This work validates the scalability of organoid cocultures for large‐scale drug discovery campaigns, bridging the gap between basic biology and clinical drug development. Based on the high‐content imaging platform established by Cattaneo et al. [37] for visualizing T‐cell–organoid interactions, recent studies have applied this technology to large‐scale drug screening. For instance, Zhou et al. [151] utilized this coculture system to screen epigenetic libraries, successfully identifying inhibitors that upregulate MHC‐I expression and sensitize refractory tumor organoids to T‐cell‐mediated cytotoxicity.

In summary, integrating TME components overcomes the physiological limitations of conventional models. These systems serve as a rigorous filter for drug candidates, effectively bridging the gap between preclinical screening and clinical reality. Currently, the clinical utility of organoid cocultures is being actively evaluated in registered trials. A notable example is NCT06787625, which focuses on developing organoids from primary colorectal tumors and liver metastases. Distinctively, this study incorporates functional coculture assays using autologous PBMCs to recreate the patient‐specific immune microenvironment. This approach is designed not only to test existing drugs but to uncover novel therapeutic vulnerabilities and signaling pathways amenable to targeted therapies and immunotherapies, thereby refining the precision oncology pipeline.

### 5.2 PD‐1/PD‐L1 Immunotherapy

Immune checkpoint inhibitors, particularly PD‐1/PD‐L1 blockers, stand out as a prime illustration of groundbreaking advancements [152]. Since the approval of the first immune checkpoint inhibitor, ipilimumab, by the FDA in 2011 for the treatment of metastatic melanoma, immunotherapy—especially PD‐1/PD‐L1‐targeted treatments—has played an increasingly significant role in managing various solid tumors [153]. However, only a subset of “immune‐hot” tumors respond to PD‐1/PD‐L1 therapies resulting frequently suboptimal treatment outcomes. Also, there are currently no definitive biomarkers to predict individual responses to these treatments. Therefore, predicting therapeutic efficacy and investigating the mechanisms of resistance to immunotherapy have become key research priorities [154].

Mucosal melanoma represents as an aggressive subtype of melanoma, often associated with unfavorable prognoses. Transcriptome analysis has unveiled a noteworthy finding: receptor tyrosine kinases (RTKs) signaling, notably nerve growth factor receptor (NGFR), exhibits significant upregulation in oral mucosal melanoma organoids from patients resistant to anti‐PD‐1 therapy. Moreover, coculture systems integrating oral mucosal melanoma organoids with autologous CD8+ T cells or PBMCs have demonstrated that interventions such as anlotinib treatment or NGFR knockdown in mucosal melanoma organoids markedly augment the efficacy of immune cells in combating PD‐1‐resistant oral mucosal melanoma under nivolumab therapy [155]. These findings underscore the pivotal role of the RTK family, particularly NGFR, in potentially driving resistance to anti‐PD‐1 therapy in oral mucosal melanoma cases. Immune therapies have shown limited efficacy in addressing high‐grade serous ovarian cancer (HGSC). Wan et al. [156] undertook immune functional analyses and single‐cell RNA‐seq transcriptional profiling using HGSC organoid/immune cell coculture. These coculture systems were exposed to a unique bispecific anti‐PD‐1/PD‐L1 antibody, facilitating comparison with monospecific anti‐PD‐1 or anti‐PD‐L1 controls. The outcome shows that by downregulating BRD1 expression in immune cells, the bispecific antibody (BsAb) increased cytotoxicity in T and NK cells while decreased exhaustion in T cells induced most strongly. BRD1 inhibition similarly enhanced the effectiveness of immune checkpoint therapy, which enlightens the possibility of combination therapy.

Besides, elevated serum Dickkopf 1(DKK1) was reported to predicted poor tumor response to PD‐1 blockade in CRC with deficient mismatch repair (dMMR) or microsatellite instability (MSI). It exerts its inhibitory effects on antitumor immune responses through the GSK3β/E2F1/T‐bet axis, ultimately leading to the inactivation of CD8+ T cells. In a coculture model of dMMR CRC organoids with CD8+ T cells, simultaneous neutralization of both PD‐1 and DKK1 resulted in a notably higher proportion of apoptotic tumor cells, compared to the treatment with either PD‐1 or DKK1 alone [42]. This underscores the potency of DKK1 neutralization in augmenting the antitumor response of dMMR/MSI CRC cells to PD‐1 blockade. Overall, these findings suggest that resistance to immunotherapy could not only from tumor cell genomic reasons but also from factors within the solid TME, contributing to dysfunction among key immune cells like CD8+ T cells and NK cells. To conclude, in the background of organoid immune coculture, study of PD‐1/PD‐L1 drug resistance proves invaluable for unraveling the mechanisms behind resistance and advancing immune combination therapy.

While the majority of PD‐1/PD‐L1 screening studies utilizing organoid cocultures remain in the preclinical phase, the transition to clinical validation is underway. Trials in solid tumors, such as glioblastoma (NCT06781372), are pioneering the use of “immune‐enhanced” organoids to predict responses to checkpoint blockade. These studies represent a critical step toward the ultimate goal: interventional trials where organoid–immune coculture data directly guides the prescription of PD‐1/PD‐L1 inhibitors in personalized oncology.

### 5.3 CAR‐Engineered Lymphocytes Immunotherapy

CAR‐based therapy represents a paradigm shift in oncology, allowing immune cells to recognize surface antigens without MHC restriction. While initially T‐cell centric, the field is rapidly diversifying to include innate effectors such as NK cells and macrophages. In recent years, chimeric antigen receptor T‐cell (CAR‐T) immunotherapy is a very promising scheme in tumor immunotherapy, and it has emerged in the treatment of a variety of hematological tumors [157]. However, CAR‐T therapy still faces many problems such as high cost, low production efficiency and instability, and limited dose, which limit its application [158]. Coculture models offer an ideal platform for studying the application of CAR‐T therapy in tumors. On one hand, coculturing tumor organoids with relevant stem cells enables the production of CAR‐T cells. On the other hand, immune coculture models can be utilized to validate the antitumor efficacy of CAR‐T cells. Christine's team findings suggest that by reprogramming CD62L+ naive and memory T cells into induced pluripotent stem cells (iPSCs), then engineering them with CD19‐CAR and differentiating them in a 3D organoid system, they obtained products exhibiting similar characteristics to conventional CD8αβ‐positive CAR‐T cells. The expanded iPSC induced CD19 CAR‐T cells displayed comparable antigen‐specific activation, degranulation, cytotoxicity, and cytokine secretion to conventional CD19 CAR‐T cells. Moreover, they maintained consistent expression of the T‐cell receptor (TCR) derived from the original clone [159]. 3D organoid coculture is critical in this process, as the iPSC CAR‐T cells produced using a monolayer coculture system exhibited characteristics resembling innate immune cells (specifically, CD8αα+ phenotype). Additionally, they demonstrated lower levels of antigen‐specific cytotoxicity and cytokine secretion when compared to conventional CAR T cells [160].

To demonstrate the potential of CD39 as a marker for identifying active antitumor immune cells relevant to cellular immunotherapy, an HCC organoid–T‐cell coculture system was built. This system was utilized to assess the effectiveness of CD39+ HBVs‐CAR‐T cells and CD39+ personalized tumor‐reactive CD8+ T cells. In this research, T cells sourced from PBMCs were engineered into HBVs‐CAR‐T cells through CAR transduction and HCC organoid coculture. Concurrently, personalized tumor‐reactive CD8+ T cells were cultured alongside autologous tumor organoids. In both modified cell types, the CD39+ subsets demonstrated superior antitumor activity [161]. These findings imply that CD39 holds significant promise as a marker in immunotherapy. For instance, CD39+CD8+ T cells could represent a pivotal subpopulation in cellular immunotherapy for HCC treatment. By enriching and engineering CD39 positive T cells, we may enhance the efficacy of immunotherapeutic interventions.

While CAR‐T‐cell therapies have transformed the landscape of hematologic malignancies, their widespread application is limited by the need for autologous manufacturing and the risk of severe toxicities such as cytokine release syndrome (CRS) and neurotoxicity. In contrast, CAR‐engineered natural killer (CAR‐NK) cells have emerged as a promising allogeneic solution. Unlike T cells, NK cells do not induce graft‐versus‐host disease (GVHD), allowing for the development of “off‐the‐shelf” products derived from peripheral blood, umbilical cord blood, or continuously expanding cell lines like NK‐92 [162]. Mechanistically, CAR‐NK cells possess a unique “dual‐killing” capability. Beyond the antigen‐specific recognition mediated by the CAR, they retain their innate cytotoxicity triggered by natural cytotoxicity receptors like NKp30, NKp44 and the downregulation of MHC Class I molecules on tumor cells. This intrinsic ability enables CAR‐NK cells to eliminate heterogeneous tumors and mitigate relapse caused by antigen escape, a common failure mode in CAR‐T therapy [163]. For successful clinical translation, accurately predicting CAR‐NK safety and potency is paramount. Here, a 3D coculture assay pairing CAR‐NK cells with PDOs served as a robust translational platform. By enabling the comparative analysis of cytotoxicity against both tumor and matched healthy organoids, this system identified potential safety hazards in novel targets (FRIZZLED) while confirming the precision of others (EGFRvIII), proving its value as a reliable predictive tool for precision oncology [164].

Building upon preclinical foundations, recent clinical initiatives are validating organoid cocultures as predictive tools to guide patient care. The shift toward active clinical decision support is exemplified by the TargetCRC trial (NCT05401318) in CRC. This study utilizes a functional ex vivo pipeline to test combinatorial strategies, exposing PDOs to CAR‐T cells alongside chemotherapy to identify synergistic mechanisms that overcome tumor resistance. Similarly, the CARMA study (NCT05007379) validates the use of organoids for assessing CAR‐Macrophages (CAR‐M) in breast cancer, establishing a potency assay that accounts for the stromal penetration advantages of innate immune cells. Crucially, the NCT07153289 trial expands the utility of these platforms by integrating organoid cocultures into a translational framework for CD318‐CAR‐T cells in PDAC. Here, organoid models are employed alongside single‐cell multiomics to dissect treatment resistance and TME changes, identifying immunological correlates that guide the optimization of next‐generation CAR constructs. Collectively, these efforts highlight the indispensable role of organoid cocultures in derisking and refining CAR‐engineered lymphocytes immunotherapy for precision oncology.

### 5.4 Vaccines

In addition to PD‐1 blockade and CAR‐engineered lymphocytes therapy as discussed earlier, coculture models serve as invaluable tools for investigating various other immune‐related therapies. Cancer vaccines are immunotherapeutic agents that aim to elicit or amplify antigen‐specific immune responses against malignant cells. Unlike prophylactic vaccines that prevent infection‐related cancers, therapeutic cancer vaccines leverage tumor‐associated antigens to prime antigen‐presenting cells and activate cytotoxic T lymphocytes and helper T cells [165, 166]. Typically, DC‐based vaccines method is one of the key component [167]. HELA‐Exos represents as an in situ DC vaccine designed to boost the antitumor response in breast cancer. Following administration to organoid–PBMC coculture systems, HELA‐Exos demonstrated enhanced cross‐presentation of conventional dendritic cell type 1 (cDC1) antigens and generation of tumor‐reactive CD8+ T cells [36]. The immunomodulatory impact of this in situ vaccine was verified in vitro through coculture models. To overcome the challenge of immune‐cold tumors, Shang et al. [168] developed a strategy using organoid‐pulsed nanocomplexes. In this approach, PDOs served as a high‐fidelity antigen source to load DC‐derived microvesicles. The resulting nanovaccine successfully promoted DC migration and turned “cold” tumors “hot,” leading to extended survival in in situ pancreatic and lung cancer studies. These results underscore a paradigm shift where organoids are utilized directly as functional components in vaccine engineering rather than solely for efficacy testing.

Some clinical trial designs for cancer vaccines have also incorporated coculture models. In the NCT06156150 trial, a PDO platform is employed to evaluate the probability of reversing resistance to DC vaccines. By coculturing autologous organoids with immune effectors, the study investigates the immunosuppressive B7‐H4 axis, revealing its role in dampening T‐cell recruitment via the downregulation of CXCL9/10 chemokines. This “bedside‐to‐bench” approach not only confirms the mechanism of vaccine failure but also establishes a predictive model. The data derived from these coculture experiments are intended to stratify patients based on B7‐H4 expression and clinicopathologic features, ultimately guiding the development of combination strategies that can sensitize “cold” gliomas to vaccine‐induced immunity. Similarly, in the context of lynch syndrome, the Nous‐209 vaccine trial (NCT05078866) leverages organoid technology to validate cancer interception strategies. By establishing cocultures of patient T cells and matched colorectal adenoma organoids, the study functionally verifies whether the vaccine‐induced immune response translates into effective cytotoxicity against precancerous polyps.

Taken together, these findings underscore the versatility of coculture platforms, which serve as a comprehensive toolset for both identifying immunogenic antigens and validating the functional potency of cancer vaccines.

### 5.5 Bispecific Antibodies

BsAbs are engineered proteins that can simultaneously bind two distinct antigens or two epitopes, enabling novel therapeutic mechanisms not achievable by conventional monoclonal antibodies. These mechanisms include redirecting immune effector cells to tumor cells, dual signaling pathway blockade, and recruitment of innate immune cells. BsAb formats range from IgG‐like to non‐IgG architectures optimized for pharmacokinetics and tissue penetration. This dual targeting enhances tumor specificity and can overcome biological redundancy of single targets [169]. While BsAbs have traditionally excelled in hematologic cancers, clinical evidence is emerging for solid tumors [170, 171]. A representative example is ivonescimab, a PD‐1 × VEGF BsAb, which harnesses dual mechanisms of immune activation and antiangiogenesis. Ivonescimab is currently being evaluated in a pivotal phase III trial (NCT06767527) as a first‐line therapy for triple‐negative breast cancer (TNBC). It is expected to become the first dual‐target immunotherapeutic antibody in China with potential application in the treatment of TNBC. Collectively, these findings underscore that BsAbs represent a pivotal frontier in the future of cancer therapy.

In this context, organoid coculture systems precisely serve as an ideal platform for evaluating the efficacy of BsAbs. BsAbs that bind to a surface tumor antigen and CD3ε trigger T‐cell‐mediated killing of tumors [172]. Using live confocal microscopy, 3D organoid–T‐cell cultures are effective models to test how CEA‐CD3 T‐cell engagers treatments kill colon cancer organoids. The results indicate that the levels of CEA expression determine how effectively T‐cells can kill cancer cells [173, 174]. Hence, colon cancer patients should undergo screening for high mRNA expression of CEA before participating in current clinical trials (NCT02324257), in which the CEA‐CD3 T‐cell BsAb cibisatamab is tested. In another study, researchers utilized a coculture system comprising PBMCs and HER2+ gastric cancer PDOs to dissect the resistance mechanisms to anti‐HER2 monoclonal antibodies [175]. This model was instrumental in identifying that T‐cell‐derived IL‐2 is essential for sustaining NK cell‐mediated antibody‐dependent cellular cytotoxicity (ADCC). Importantly, the study employed this coculture platform to validate a combinatorial strategy: the addition of a HER2 × CD3 BsAb. The model successfully demonstrated that the BsAb could activate T cells within the microenvironment, thereby restoring the compromised ADCC response and enhancing the overall therapeutic efficacy of anti‐HER2 mAb treatment. Beyond conventional T cells, γδ T cells are also emerging as targets for bispecific engagement. Following 20 years of preclinical investigations and initial clinical trials with limited patient cohorts, γδ T cells are now emerging as a viable and promising avenue for cancer immunotherapy [176]. To establish an in vitro model for in‐depth analysis, Dong et al. [177] cocultured cervical cancer organoids with in vitro expanded Vγ9Vδ2 γδ T cells isolated from healthy donors. The improved elimination of HPV‐transformed and cancer‐derived organoids is associated with the involvement of BTN‐family members BTN2A1/3A1 in γδ T‐cell‐mediated cytotoxicity. Understanding these mechanisms suggests the potential to enhance the antitumor effects of γδ T cells through the use of agonistic anti‐BTN3A1 antibodies or bispecific γδ T‐cell engagers.

In summary, these studies demonstrate that organoid cocultures are essential tools for validating the efficacy of novel BsAbs, serving as a vital bridge between preclinical discovery and successful clinical translation.

### 5.6 Physical Therapies

Tumor physical therapies constitute a major class of locoregional treatment methods, which eradicate malignant tissue through physical rather than purely pharmacologic mechanisms. Among them, radiotherapy remains a cornerstone of cancer management, achieving cytotoxicity primarily through DNA double‐strand break induction, free‐radical generation, and mitotic catastrophe, with well‐established dose‐fractionation principles and normal‐tissue response models underpinning its clinical use across solid tumors [178, 179]. For example, an excellent research indicates that PDOs can function as predictive surrogates for neoadjuvant chemoradiation response in locally advanced rectal cancer [180]. The living biobank of rectal cancer organoids was established from patients with locally advanced rectal cancer, and the models retained the histopathological and genomic features of the corresponding tumors. Notably, ex vivo chemoradiation responses showed a strong concordance with clinical outcomes (accuracy 84.43%, sensitivity 78.01%, specificity 91.97%), supporting the potential of PDO‐based assays as companion diagnostic tools to guide patient stratification and personalize radiotherapy strategies. Similarly, in a study focused on the BRAF V600E mutation, BRAF V600E mutant PDOs served as a critical platform to assess therapeutic vulnerabilities [181]. The integration of genomic profiling with radiosensitivity assays revealed that BRAF V600E confers resistance to radiation. Crucially, the concordance observed between preclinical data and actual patient outcomes underscores the potential of this platform to guide precise radiotherapy decision for BRAF‐mutant patients. Currently, numerous clinical trials utilizing PDO‐based models for radiotherapy response prediction are underway, notably in breast cancer (NCT06468124), cervical cancer (NCT06786780), rectal cancer (NCT03577808). Taking together, these studies underscore the robust capability of organoid models to simulate radiotherapy‐induced tumor killing.

Cryoablation, a treatment involving extreme cold, induces necrosis and apoptosis in cells, offering a less invasive alternative to conventional surgical resection. The necrosis triggered by extreme cold has the potential to stimulate systemic antitumor immunity by releasing tumor antigens [182, 183]. Enhanced antitumor effects were observed when organoids were cocultured with autologous lymphocytes harvested after cryoablation to bladder cancer comparing to prefreezing autologous lymphocytes. Besides, experiments based on coculture models suggest that IFNGR expression in tumors is necessary for cryoablation to induce antitumor immunity and is linked to improved prognosis following cryoablation [184]. These findings offer foundational evidence supporting the application of cryoablation, with potential for integration alongside other immunotherapies. The strong predictive value evidenced by the above‐mentioned coculture and clinical data supports the effective implementation of coculture models in tumor physical therapies.

### 5.7 Antibody Drug Conjugates

Antibody–drug conjugates (ADCs) have emerged as a powerful type of therapeutics by delivering cytotoxic payloads directly to tumor cells by antigen‐specific antibodies. However, the efficacy of ADCs relies heavily on complex spatial mechanisms—such as intratumoral penetration, internalization, and the “bystander killing effect”—which are poorly recapitulated in monolayer models [185, 186]. Organoid models bridge this gap by preserving the 3D architecture and heterogeneity of the parental tumor. Unlike 2D models, organoids allow researchers to evaluate the penetration depth of large ADC molecules within dense tumor tissues. More importantly, organoids are the ideal platform to validate the bystander effect, where a permeable cytotoxic payload released from an antigen‐positive cell kills surrounding antigen‐negative cells.

A recent study utilized a coculture system of PDOs and CAFs to evaluate 84‐EBET, a novel CEACAM6‐targeting ADC equipped with a BET protein degrader [187]. They first conducted compound screening using an organoid coculture system, through which they identified EBET‐1055, a BET protein degrader exhibiting strong efficacy in PC‐3 and PC‐42 organoids, as the optimal payload candidate from their proprietary compound library. Next, using a tumor organoid–CAF coculture model, they demonstrated that EBET induces cytotoxicity in PDAC cells through BET degradation, while simultaneously modulating the inflammatory and protumorigenic activity of CAFs. These findings indicate that the payload not only targets tumor cells but also reprograms stromal components, and its effects on CEACAM6‐negative cells and CAFs further reflect a pronounced bystander effect. In another study, the authors established organoid and coculture models to identify and validate a therapeutic target and cytotoxic payload for bladder cancer [188]. Using PDOs and in vivo models, they identify 7‐ethyl‐9‐fluorocamptothecin (A2), a camptothecin derivative with robust antitumor activity across PDO, CDX, and PDX systems. Mechanistic pull‐down and mass spectrometry analyses reveal MAD2L1 as the direct molecular target of A2, with binding at Lys73 leading to activation of the cGAS–STING pathway and induction of apoptosis in bladder cancer cells. To overcome the dose‐limiting toxicity associated with insufficient tumor selectivity, the authors develop LZU‐WZLYCS01, an FGFR3‐targeted ADC using A2 as the payload. The ADC demonstrates FGFR3‐dependent activity, reduced efficacy in FGFR3‐knockout models, and strong tumor‐selective accumulation in vivo. Notably, organoid coculture models confirm a pronounced bystander effect, while PDO and CDX/PDX evaluations show potent antitumor efficacy with a favorable safety profile. Moreover, LZU‐WZLYCS01 outperforms gemcitabine cisplatin and retains activity in GC‐resistant PDX and PDO models. These findings highlight the value of organoid coculture models guided target and payload discovery for advancing next‐generation ADC therapy in drug‐resistant cancer.

Clinical studies integrating tumor organoid–ADC coculture are also progressing steadily. A notable example of clinical translation is the randomized Phase II ORIENTA trial (NCT06102824), which evaluates the efficacy of organoid‐guided treatment (OGT) compared to physician's choice treatment. Crucially, the drug screening library in this study incorporates major ADCs, including Sacituzumab govitecan (targeting TROP2) and Trastuzumab deruxtecan (targeting HER2, including low‐expression cases). This study represents one of the few pioneering clinical trials where organoid drug sensitivity profiling is used to directly dictate the clinical administration of ADCs to patients.

### 5.8 New Platforms With Analytics

Due to the complex 3D architecture of organoids, the accurate assessment and quantification of organoid number and drug cytotoxicity have remained a persistent challenge. However, emerging platforms integrated with advanced analytics have recently significantly facilitated the clinical translation of organoid coculture models.

Quantification using the CellTiter‐Glo 3D Cell Viability Assay is the conventional approach for assessing organoid viability. Nevertheless, this lytic assay destroys organoid architecture, thereby precluding the acquisition of morphological data [189]. By leveraging a combination of Z‐stack microscopy and fluorescent markers, Hua's team established a comprehensive platform for 3D organoid analysis [190]. This methodology allows for the temporal tracking of size changes and the assessment of global well readouts, thereby revealing distinct sensitivity profiles to drug treatments. Ramos Zapatero et al. [191] have developed highly multiplexed single‐cell post‐translational modification (PTM) profiling using TOBis mass cytometry, and tree‐based treatment effect analysis via Trellis, which can unveil patient‐specific drug responses in thousands of PDO–CAF cultures. Its identification of CAF‐driven stem‐cell state plasticity as a mediator of drug tolerance offers actionable targets and supports the development of rational combination therapies for clinical translation. Artificial intelligence (AI) and machine learning are advancing rapidly, and the data generated from coculture experiments can likewise be analyzed using relevant algorithms. Guinn et al. [192] utilized computational validation to confirm that CAFs induce inflammatory and EMT states in epithelial cells. They employed transfer learning methods and analyzed transcriptional data obtained from PDOs and cocultures with CAFs. Moreover, large vision models (such as SAM2) combined with image‐analysis algorithms enable unsupervised segmentation, dynamic tracking, and quantitative feature extraction from time‐series images [193]. They made measurement of organoid volume, growth rate, and morphological or pathological dynamics. The application of these new platforms with analytics will facilitate the better use of coculture models in translational medical research and compensate for the defects.

In summary, the efficacy of precision oncology, ranging from ADCs and immunotherapies to physical interventions, depends heavily on the TME. By integrating TME components, organoid cocultures offer superior predictive accuracy compared to simple monocultures. These models successfully capture complex mechanisms, such as ADC bystander effects and immunogenic responses. Relevant clinical trials have been summarized in Table 2: Clinical Trials Investigating Organoid Cocultures in New Therapies. To further translate these findings into a practical framework, Table 3 provides a structured “bench‐to‐clinic” case matrix. This matrix aligns various treatment modalities with their respective coculture models, mechanistic findings, and potential biomarkers, offering a reusable guide for advancing personalized therapeutic strategies from the laboratory to the clinic. However, increased biological complexity inevitably brings technical variability. Therefore, the following section will discuss the critical challenges regarding standardization and future perspective facing the field.

**TABLE 2:** Clinical trials investigating organoid cocultures in new therapies.

NCT number	Disease type	Status	Study objectives
NCT06787625	Colorectal cancer	Recruiting	To establish PDOs coculture with PBMCs and evaluate feasibility in predicting immune therapy response.
NCT06781372	Glioblastoma	Not yet recruiting	Assessing the potential of PDO‐immune cocultures to predict PD‐1/PD‐L1 inhibitors response.
NCT05401318	Colorectal cancer	Recruiting	Prospective comparison of PDO CAR‐T sensitivity results with clinical patient responses.
NCT05007379	Colorectal cancer	Unknown status	Predicting the efficacy of CAR‐Macrophages in locally advanced CRC.
NCT07153289	Pancreatic cancer	Not yet recruiting	Integrating organoid cocultures into a translational framework for CD318‐CAR‐T cells in PDAC.
NCT06156150	Glioma	Recruiting	Investigating how B7‐H4 drives chemokine‐mediated T‐cell exclusion and vaccine resistance.
NCT05078866	Lynch syndrome	Active, not recruiting	Evaluating whether the vaccine‐induced immune response translates into effective cytotoxicity against precancerous polyps.
NCT06767527	Triple‐negative breast cancer	Not yet recruiting	To evaluate Ivonescimab as a first‐line therapy for triple‐negative breast cancer.
NCT02324257	Multiple solid tumors	Completed	To test safety, pharmacokinetics, and therapeutic activity of CEA‐CD3 T‐cell bispecific antibody‐Cibisatamab.
NCT06468124	Breast cancer	Recruiting	Evaluating sensitivity of organoids to predict treatment outcome in breast cancer metastases.
NCT06786780	Cervical cancer	Enrolling by invitation	Assessing the efficacy of organoids in predicting the response to radiotherapy and chemotherapy in cervical cancer.
NCT03577808	Rectal cancer	Unknown status	To validate whether the organoids could predict the clinical outcome in locally advanced rectal cancer.
NCT06102824	Breast cancer	Recruiting	To evaluate the efficacy of organoid‐guided treatment compared to physician's choice treatment.

*Data sources*: Clinical registration website (ClinicalTrials.gov).

Abbreviations: CAR‐T, chimeric antigen receptor T‐cell; CEA, carcinoembryonic antigen; CRC, colorectal cancer; PBMCs, peripheral blood mononuclear cells; PDAC, pancreatic ductal adenocarcinoma; PDOs, patient‐derived organoids.

**TABLE 3:** A bench‐to‐clinic matrix of organoid coculture applications across diverse therapeutic modalities.

Strategy	Coculture model	Main mechanistic finding	Potential biomarkers	Clinical implication	References
High‐throughput screening	PDOs + Vast drug libraries	Facilitates large‐scale discovery of novel antitumor compounds.	Drug sensitivity IC50	Identifying lead compounds for rare or refractory cancers.	[150, 151]
PD‐1/PD‐L1 inhibitors	PDOs + CD8+ T cells/PBMCs + anti‐PD‐1/PD‐L1 therapy	RTK family signaling drives resistance to anti‐PD‐1 therapy.	RTK expression	Stratifying patients for anti‐PD‐1/RTK family combination therapy.	[155, 156]
CAR‐engineered cells	PDOs + CAR‐X	Validates CAR‐based cell killing; identifies markers for active immune cells.	CD39; CAR persistence	Utilizing organoids to evaluate the efficacy of CAR therapies.	[161, 164]
Cancer vaccines	PDOs + PBMCs + Vaccines	Verifies immunomodulatory impact of DC‐based vaccines on the TME.	Cytokine profiles	Evaluating vaccine‐induced T‐cell activation in patient‐specific models.	[36, 168]
Bispecific antibodies	PDOs + T cells/PBMCs + bispecific antibodies	T‐cell‐derived IL‐2 sustains NK‐mediated ADCC; efficacy depends on antigen levels.	CEA expression levels; IL‐2 concentration	Optimization of bispecific antibody dosing and target selection (CEA).	[173, 174, 175]
Radiotherapy	PDOs + Radiotherapy	PDOs predict neoadjuvant chemoradiation response; mutations influence radiotherapy resistance.	BRAF V600E mutation	Personalizing radiation dose and identifying candidates for radio‐sensitizers.	[181]
Cryoablation	PDOs + Autologous lymphocytes harvested after cryoablation	IFNGR expression is essential for cryoablation‐induced antitumor immunity.	IFNGR expression	Predicting long‐term prognosis following ablation combined with immunotherapy.	[184]
ADCs	PDOs + CAFs	Coculture models guide target selection and payload discovery against stromal barriers.	Target antigen density; Stromal markers	Advancing next‐generation ADCs to overcome TME‐mediated resistance.	[187]

Abbreviations: ADCC, antibody‐dependent cellular cytotoxicity; ADCs, antibody–drug conjugates; CAFs, cancer‐associated fibroblast; CAR, chimeric antigen receptor; CEA, carcinoembryonic antigen; DC, dendritic cells; NK, natural killer; PBMCs, peripheral blood mononuclear cells; PDOs, patient‐derived organoids; RTK, receptor tyrosine kinases; TME, tumor microenvironment.

## 6 Current Challenges and Future Perspectives

Coculture models serve as essential tools for not only studying TME interactions but also advancing clinical therapies translation. Nevertheless, coculture models have certain limitations that hinder the broader application. The first problem is that current coculture models often involve only one or two components of the microenvironment, which does not capture its full complexity. One important reason is that during the primary generation of tumor organoids, other TME components, such as immune cells and blood vessels, gradually disappear. Also, current coculture models represent isolated systems that lack systemic integration. Notably, the absence of hepatic metabolism limits the evaluation of prodrugs and drug pharmacokinetics [194]. Furthermore, without a functional circulation and lymphatic system, these models fail to capture the dynamic recruitment and infiltration of immune cells from peripheral tissues [195]. These constraints hamper the utility of coculture models as preclinical tools for mimicking the intricate in vivo microenvironment, thereby impeding their effectiveness in elucidating mechanisms, screening drugs, and predicting efficacy models. Therefore, coculture is needed to reintroduce these cells and better simulate the microenvironment. Nevertheless, introducing diverse TME elements often compromises the stability of organoid cultures. While strategies such as ALI or microfluidic platforms, as highlighted in Section 3, can partially alleviate these challenges. Hence, striking the right balance between components is critical for model fidelity.

Equally important is the issue of standardization in organoid establishment. Compared to well‐established mouse models and 2D cell lines, the standardization of organoids remains to be improved. First, the majority of tumor organoids rely on primary PDO culture; consequently, there are no standardized, long‐term passing organoid lines for stable expansion and research. Second, as outlined in Section 4.7, the experimental reproducibility is often challenged by the mouse derived nature of the matrix and its associated batch variability [196]. Similarly, despite recent efforts to standardize the complex culture media, variations in growth factor additives continue to be a source of heterogeneity. Furthermore, organoids often exhibit diverse morphologies and sizes during culture, predominantly appearing as cystic or solid structures [197]. The opacity and density of organoids restrict imaging depth and impede automated quantitative analysis. This heterogeneity complicates the quantification of organoid size and number.

High cost and technical expertise are also challenges. Maintaining organoid coculture models requires specialized skills, equipment, making it inaccessible to some research teams. The establishment of PDOs is a lengthy process, typically requiring a minimum of 1 week, and is fraught with risks such as organoid apoptosis, contamination, and establishment failure [198]. The protracted timeline required to establish and expand PDOs often conflicts with the urgent clinical need for therapeutic decision‐making in advanced cancer patients. Furthermore, the essential reagents for organoid maintenance, specifically Matrigel and recombinant growth factors, remain relatively expensive. This renders the approach less economically viable and, to some extent, hinders the widespread adoption of organoid models [31]. Another significant challenge encountered in utilizing 3D organoid coculture models for research is the frequent absence of validated assessment tools. Evaluation techniques commonly employed in 2D cultures may not be suitable for 3D culture systems due to their distinct construction methods and growth characteristics of cell clusters [199]. Besides, utilizing organoids derived from patient samples may raise ethical and regulatory concerns regarding patient consent, privacy, and the ownership of biological materials.

Despite these challenges, we still remain convinced of the immense potential of organoid models in translational medicine. Furthermore, with the recent integration of emerging technologies, these challenges are being progressively overcome. Future advancements will likely converge on engineering reliability, increasing biological complexity, and enhancing clinical translation.

To transition organoid coculture models from academic concept to robust industrial tools, engineering reliability is paramount. Future efforts must focus on reducing experimental variability through key strategies. Shifting from undefined animal‐derived Matrigel to chemically defined synthetic hydrogels will be essential. As mentioned in Section 4.7, these tunable materials not only eliminate batch‐to‐batch variations but also allow for the precise recapitulation of tissue‐specific mechanical properties like stiffness. Furthermore, there is a critical need to refine culture protocols to ensure the standardization and reproducibility of organoid systems. Future efforts should also explore the feasibility of establishing stable, long‐term organoid lines to meet the demands of drug screening and experimental research.

Increasing the biological complexity of organoid coculture models relies heavily on the advancement of emerging technologies. The 3D bioprinting and microfluidic models mentioned in Section 3.4 represent a breakthrough from traditional coculture frameworks and prove highly effective in certain specialized application situations. They enable precise spatial organization, dynamic perfusion, and multibiological components that are not achievable in conventional organoid coculture models. Microfluidic “organ‐on‐a‐chip” architectures introduce physiologically relevant flow, biochemical gradients, and mechanical cues, thereby supporting long‐term coculture of epithelial, stromal, immune, and vascular compartments while preserving functional crosstalk and emergent microenvironmental heterogeneity [200]. In parallel, 3D bioprinting provides deterministic control over cell type patterning, ECM composition, and tissue geometry, allowing the assembly of hierarchically organized organoid‐based tissues that recapitulate structural features such as zonation, vascular proximity, and tumor–stroma interfaces [201]. We anticipate that, as technological advances continue to reduce costs, the integration of microfluidic platforms and 3D bioprinting will propel organoid coculture systems into an future of automated and mechanized cultivation.

More importantly, organoid coculture models are expected to play an increasingly critical role in enhancing clinical translation from bench to bedside in the future. With regulatory milestones such as the FDA Modernization Act 2.0, which endorses nonanimal alternatives, the path for organoids to become a standard tool in drug development and precision medicine is clearer than ever [202]. Organoid biobanks enable clinical Phase 0 trials in a dish, facilitating the early attrition of nonefficacious drugs and reducing animal reliance [203]. Given their superior negative predictive value compared to efficacy prediction, future clinical strategies will prioritize using organoids to rule out ineffective therapies [33]. This approach minimizes unnecessary toxicity and preserves the patient's critical therapeutic window. Organoids have become a staple in HTS for chemotherapy and targeted drugs. Notably, the establishment of coculture systems has opened new avenues for screening immuno‐oncology agents, BsAbs, and physical therapies. We expect a surge in clinical trials dedicated to validating these emerging therapies using organoid coculture platforms. Furthermore, the patient‐specific characteristics of PDOs position them as a cornerstone of precision medicine. The anticipated launch of coclinical trials, which run parallel to human trials, will be pivotal in verifying the capability of organoids to predict clinical responses. Addressing the critical issue of drug resistance, we envision a workflow where PDOs derived from surgically resected tissue are used to pre‐emptively evaluate efficiency and side effects of the therapies. This would allow for the data‐driven selection of therapies, ensuring that every patient receives a unique treatment strategy aligned with the principles of precision medicine.

## 7 Conclusions

To conclude, organoid coculture models have emerged as indispensable tools for mimicking the architectural and functional complexity of the TME in vitro. As summarized above, diverse methodologies, from direct coculture and Transwell systems to microfluidics and 3D bioprinting, offer versatile platforms to explore TME crosstalk mediated by direct contact or secreted factors. Beyond mechanistic studies, these models are increasingly important in clinical settings. We have outlined the expanding role of organoids in evaluating emerging therapies, with a growing presence in clinical trials involving ADCs, immunotherapies, and physical therapeutic methods. Nevertheless, the transition from laboratory to clinic is still facing problems. Issues regarding standardization, reproducibility, and cost‐effectiveness represent significant barriers. Looking forward, the convergence of organoid technology with emerging innovations, such as microfluidics, high‐content imaging, and AI‐driven analytics, promises to overcome these limitations. Ultimately, the establishment of standardized, patient‐specific coclinical and preclinical trials will be instrumental in realizing the goal of precision medicine, enabling rational drug selection and improving clinical outcomes for cancer patients.

## Author Contributions

Junjie Peng, Shaobo Mo, and Hai Zou had the idea of this article, Jiajun Yang and Chunliang Cheng drafted the manuscript and prepared the figures and tables. Wenqin Luo and Xingfeng He helped collecting data and preparing the figures and tables. Yaqi Li and Xiang Hu performed the literature search and provided overall supervision. Junjie Peng, Shaobo Mo, and Sanjun Cai designed this review and revised the manuscript. All the authors contributed to the article and approved the submitted version. All the authors read and approved the final manuscript.

## Funding

This work was supported by the National Natural Science Foundation of China (82203215 and 82573938 to Shaobo Mo, 82372974 to Yaqi Li, 82473500 to Junjie Peng) and Shanghai Sailing Program (22YF1408800 to Shaobo Mo).

## Ethics Statement

The authors have nothing to report.

## Conflicts of Interest

The authors declare no conflicts of interest.

## Data Availability

The authors have nothing to report.
